# A Novel Cellular Therapy for Preterm Complications: Safety Profile of Allogeneic Cord Blood Mononuclear Cells in a Pilot Clinical Study

**DOI:** 10.1155/sci/9979914

**Published:** 2026-08-12

**Authors:** Jia Chen, Yabo Mei, Liu He, Zhenlan Du, Guosheng Xing, Xue Du, Liping Cheng, Xiaofei Wei, Danhua Zhao, Yanan Gu, Qiuping Li, Zhichun Feng

**Affiliations:** ^1^ Senior Department of Pediatrics, Chinese PLA General Hospital, Beijing, China, 301hospital.com.cn; ^2^ National Engineering Laboratory for Birth Defects Prevention and Control of Key Technology, Beijing, China; ^3^ Beijing Key Laboratory of Treatment and Rehabilitation for Pediatric Critical Care, Beijing, China; ^4^ Neonatology Department, Tongzhou Maternal and Child Health Hospital, Beijing, China, pku.edu.cn; ^5^ Beijing Cord Blood Bank, Beijing, China

**Keywords:** allogenic, complication, cord blood, mononuclear cell, preterm infant, safety

## Abstract

**Background:**

Preclinical evidence supports the potential use of human umbilical cord blood‐derived mononuclear cells (hUCB‐MNCs) for preterm infants with preterm birth‐associated complications (PBAC). Allogeneic hUCB‐MNCs could extend this therapeutic option to infants without autologous cord blood available at birth.

**Methods:**

This phase I, open‐label, single‐arm, and single‐center study enrolled 10 preterm infants (gestational age [GA] 27.1 ± 0.7 weeks) with two or more PBAC. All received a single intravenous infusion of allogeneic, partially or fully human leukocyte antigen (HLA)‐mismatched hUCB‐MNCs without preconditioning or immunosuppression. The median cell was (3.51 ± 0.93) × 10^6^ cells/kg. Safety, adverse events, and clinical outcomes were assessed over a 2‐year follow‐up period.

**Results:**

All infants survived intensive care unit discharge. Four infants were lost to follow‐up for parental reasons, with no in‐hospital adverse events noted, their long‐term outcomes remain unconfirmed. No infusion‐related adverse events, acute reactions, or graft‐versus‐host disease (GVHD) were detected. One case of cerebral palsy was diagnosed during follow‐up and was unrelated to cell infusion. Five of the six infants with evaluable long‐term data had no respiratory, developmental, or neurological complications. Plasma inflammatory cytokines exhibited exploratory changes after infusion, without confirmed causality. All infants with follow‐up data reached age‐appropriate growth at 12 and 24 months of corrected age.

**Conclusions:**

No major infusion‐related adverse events were observed in preterm infants with PBAC receiving allogeneic hUCB‐MNC infusion. Six infants completed 2‐year follow‐up and four were lost to follow‐up. Constrained by small sample size and attrition, the results need further verification in larger controlled trials.

**Trial Registration:**

Chinese Clinical Trial Registry: ChiCTR‐OPN‐15006932, ChiCTR2000035227

## 1. Introduction

Preterm birth is one of the leading causes of infant morbidity and mortality worldwide [1]. Preterm infants are at a high risk for developing preterm birth complications [2]. Although current management strategies have been shown to improve most complications and overall morbidity, many survivors still face lifetime disabilities, including mental and physical motor and cognitive deficits and chronic lung disease. Therefore, developing novel and efficient therapies to reduce overall morbidity and improve the lifelong quality in preterm infants is of great clinical and social significance.

Human umbilical cord blood‐derived mononuclear cells (hUCB‐MNCs) hold considerable therapeutic potential for preterm birth‐associated complications (PBAC) [3–5], as they harbor primitive multipotent stem cells, progenitor cells and regulatory T cells that exert anti‐inflammatory and immunomodulatory effects via paracrine action and can home to damaged tissues to mediate repair [6, 7]. Preclinical studies have confirmed their potential efficacy in bronchopulmonary dysplasia (BPD) and hypoxic‐ischemic brain damage, common and severe PBAC [8–11], and autologous hUCB‐MNC infusion has been proven safe and feasible in neonates [5, 9, 12]. However, autologous cord blood is often unavailable for preterm infants (gestational age [GA] <30 weeks) due to late cord clamping, emergency deliveries, maternal pregnancy complications, and other anatomical or clinical factors, limiting the clinical application of this therapy [13–17]. While allogeneic umbilical cord blood infusion has demonstrated a favorable safety profile in prior studies, characterized by an absence of immunoreactivity, graft‐versus‐host disease (GVHD) and teratoma formation, low human leukocyte antigen (HLA) matching requirements, and no necessity for immunosuppression [18], the safety and feasibility of allogeneic hUCB‐MNC infusion in preterm infants with PBAC remain yet to be fully clarified. This study thus aimed to evaluate the short‐term and 24‐month safety and feasibility of HLA‐mismatched or partially HLA‐matched allogeneic hUCB‐MNC infusion in 10 preterm infants with PBAC.

## 2. Materials and Methods

### 2.1. Study Design

**Figure 1:**
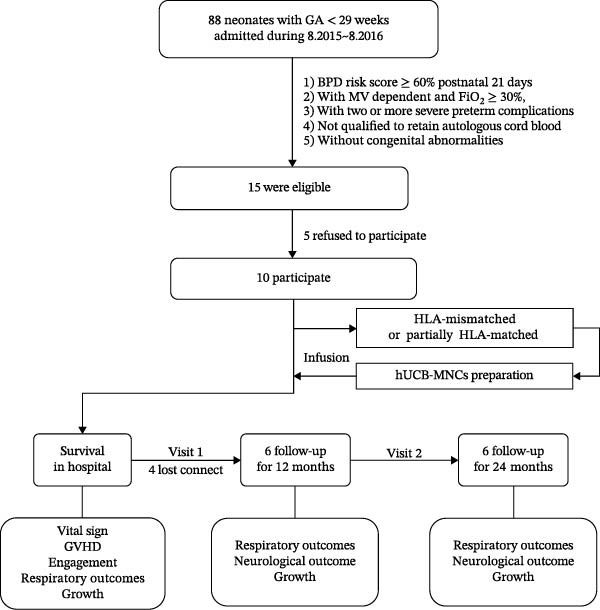
Design and processing of the study.

This study was a phase I, open‐label, single‐arm, and single‐center clinical trial. It evaluated a single intravenous infusion of volume‐reduced and red cell‐depleted allogeneic hUCB‐MNCs with full or partial HLA mismatch in preterm infants with a GA below 29 weeks and PBAC. Eligible preterm infants were enrolled only after at least two rounds of communication with their families and obtaining written informed consent from both parents. All enrolled infants received a single infusion of hUCB‐MNCs at baseline and were followed for safety assessment. No immunosuppression or conditioning was given prior to hUCB‐MNCs infusion. Additional safety endpoints were assessed at 12 and 24 months (Figure 1).

### 2.2. Patients

This pilot study was commenced in August 2015. Preterm infants admitted to the Neonatal Intensive Care Unit of the Seventh Medical Center of PLA General Hospital (Beijing, China) were eligible if they met the following criteria: (1) GA <29 weeks [13] with mechanical ventilation (MV) dependent and fraction of inspired oxygen (FiO_2_) ≥30% postnatal 20 days; (2) with two or more severe PBAC, such as BPD [19], neonatal necrotizing enterocolitis (NEC) [20, 21], ventilation‐associated pneumonia (VAP), late‐onset sepsis (LOS) [22, 23], intraventricular hemorrhage (IVH) [24], and purulent meningitis; and (3) without congenital abnormalities.

Preinfusion clinical stability and infection status were assessed per the study eligibility criteria. Notably, active sepsis, hemodynamic instability, and severe infection were permitted for enrollment rather than being defined as exclusion criteria.

The target sample size was 10 infants. The sample size was not determined by a formal statistical power calculation. All 10 enrolled infants received hUCB‐MNC infusions. No control group was included, and the study was not powered to evaluate the efficacy.

### 2.3. HLA Typing and Donor Selection

HLA typing at the HLA‐A, –B, and –DRB1 loci was performed via low‐resolution sequence‐specific oligonucleotide (SSO) typing. Donor selection followed a predefined clinical priority framework for critically ill preterm infants: (1) GA <29 weeks with severe and multiple PBAC, (2) no autologous cord blood available, (3) no 5/6 or 4/6 matched units available at the time of urgent intervention, and (4) no preconditioning or immunosuppression administered. For the 1/6 HLA‐mismatched case, the infant presented with refractory respiratory failure and multiple life‐threatening complications, and no appropriately matched cord blood units were available. This donor matching strategy was fully reviewed and approved by the Institutional Ethics Committee (No. 2015111).

### 2.4. Ethics

The study protocol and procedures were approved according to the ethical standards of the Declaration of Helsinki. The study protocol and procedures were approved by the Ethics Committee of the Seventh Medical Center of PLA General Hospital, Beijing, China on July 21^st^, 2015. The number was No. 2015111 and No. 2020‐037.

Given the small sample size and pilot nature of this study, a formal Data and Safety Monitoring Board was not constituted. Instead, safety oversight was continuously performed by the principal investigator (PI) and the Research Ethics Committees. All adverse events were reported in real‐time to the PI and the institutional review board (IRB). The PI and the Research Ethics Committees reviewed the cumulative safety data on a weekly basis to ensure participant welfare.

### 2.5. hUCB‐MNCs Preparation

hUCB was provided by the umbilical cord blood bank (Beijing, China). The cord blood samples were shipped frozen in liquid nitrogen and prepared at the Beijing Key Laboratory of Pediatric Critical Care and Rehabilitation (Beijing, China). All hUCB‐MNCs were released for infusion in accordance with NetCord‐FACT 6^th^ Edition Standards. Cryopreserved umbilical cord blood units were thawed, washed, and processed to isolate MNCs. Final hUCB‐MNCs met the following release criteria: (1) Sterility, negative for bacteria, fungi, and mycoplasma (14‐day culture method), (2) endotoxin <0.5 EU/mL (limulus amebocyte lysate assay), (3) cell viability ≥90% (flow cytometry, Beckman Coulter DxFlEX), CFU‐GM: positive colony formation (colony culture assay), and (4) DMSO residue <0.1% (high‐performance liquid chromatography), achieved by repeated washing with 0.5% human albumin during MNC processing. All final hUCB‐MNCs satisfied the release criteria prior to administration. The harvested hUCB‐MNCs were comprehensively characterized and subjected to rigorous quality control, as summarized in Supporting Information 1: Table S2 and Supporting Information 2: File 1. In brief, the total nucleated cell (TNC) count ranged from 8.18 × 10^8^ to 24.35 × 10^8^. Since granulocytes were removed via Ficoll density gradient centrifugation during isolation, TNC counts were nearly equivalent to MNC counts in the final products (MNC concentration [3.99 ± 1.01] × 10^8^/L and volume [22.55 ± 1.70] mL). All products showed satisfactory CFU‐GM activity. The infused MNCs ranged from 2.35 × 10^6^ to 5.55 × 10^6^ cells/kg. All cell products tested negative for bacterial culture, HBsAg, anti‐HCV, anti‐HIV (I/II), and syphilis, confirming microbiological safety.

### 2.6. hUCB‐MNCs Infusion

No premedication (antihistamines, steroids, or acetaminophen) was administered prior to the infusion. Vital signs including heart rate (HR), blood pressure, and oxygen saturation were continuously monitored during infusion and for 48 h thereafter, with recordings obtained every 5 min throughout the 15‐min infusion period. The cell suspension was infused at a volume of 10 mL/kg, followed by a 2 mL normal saline flush. Transient hypertension was defined as systolic blood pressure (SBP) exceeding the age‐appropriate 95^th^ percentile (80 mmHg for infants <29 weeks).

### 2.7. Outcomes

#### 2.7.1. Safety Outcomes

Safety [25] was the primary outcome including (1) monitoring vital signs before, during, and for 48 h postinfusion (HR, SBP, diastolic blood pressure [DBP], and arterial blood oxygen saturation level) [26] increase in FiO_2_ by >30%, and increase in mean airway pressure >5 cm H_2_O and (2) serial assessment of acute/chronic GVHD (rash, liver function abnormalities, diarrhea, and pulmonary complications) were performed in all infants. Donor cell chimerism was quantified via STR genotyping at 28 days after the infusion. In particular, the infant who received the 1/6 HLA‐mismatched unit underwent intensified and continuous safety monitoring throughout the study period.

Secondary outcomes included (1) successful extubation within 1 week after infusion, (2) the duration of MV with FiO_2_ ≥ 0.3 after infusion, and (3) nasal cannula (NC) flow >2 L/min, or continuous positive airway pressure (CPAP) with FiO_2_ ≥ 0.3, or noninvasive positive pressure ventilation (NIPPV) with FiO_2_ ≥ 0.3 at DOL 56 or discharge [27].

Other outcomes included the duration of hospitalization. Growth profiles included the growth curve and height curve. Laboratory examinations included white blood cell (WBC), C‐reactive protein (CRP), platelet counts (PLT), and hemoglobin (Hb). Blood gas included the potential of hydrogen (pH), partial pressure of oxygen (PO_2_), and partial pressure of carbon dioxide (PCO_2_).

#### 2.7.2. Biological Outcome

We further tested biological markers of inflammation including interleukin‐1α (IL‐1α), tumor necrosis factor‐α (TNF‐α), IL‐6, IL‐8, and IL‐10, as well as growth factors, including matrix metalloproteinase‐9 (MMP9), transforming growth factor‐β (TGF‐β), vascular endothelial growth factor (VEGF), and hepatocyte growth factor (HGF) using ELISA kits (Supporting Information 3: File 3, ELISA) before and 24 h after infusion.

#### 2.7.3. Follow‐Up Outcome

Mortality, rehospitalization, home oxygen therapy, and neuropsychological development status were assessed at 12 months and 24 months of corrected GA. Neurodevelopment was evaluated using the Gesell Developmental Schedules [28], which include five domains covering adaptive behavior, gross motor, fine motor, language, and personal‐social skills. Developmental quotient (DQ) was calculated as follows: DQ = developmental age/chronological age (corrected) × 100. DQ ≥ 85: normal, 70–84: borderline, and <70: developmental delay. All assessments were performed by trained and blinded pediatricians.

### 2.8. Statistical Analysis

Statistical methods for paired data and continuous variables. Preinfusion and postinfusion comparisons (e.g., inflammatory factors, growth factors, and blood gas parameters) were analyzed using the paired *t*‐test for normally distributed data and the Wilcoxon signed‐rank test for non‐normally distributed data. Linear mixed‐effects models were applied to analyze repeated outcome measures at four time points for continuous variables. Compound symmetry (CS) was selected as the covariance structure based on the minimum Akaike information criterion (AIC), and the model achieved good fitting. All results were expressed as mean ± standard deviation (SD) with 95% confidence interval (95% CI), and a *p*‐value < 0.05 was considered statistically significant.

## 3. Results

### 3.1. Characteristics of the Infants

Altogether 10 infants were enrolled for hUCB‐MNCs infusion from August 23, 2015 to July 31, 2016, with GA ranging from 26^+5^ to 28^+3^ (mean 27.1 ± 0.7) weeks and birth weight ranging from 730 to 1180 g (mean 1003.0 ± 154.3). Infants were dosed at a post‐natal age of 36.8 ± 14.7 days, with a range of 20–64 days. The majority of infants (*n* = 7) had maternal infection during pregnancy. Additional characteristics of the patients are presented in Table 1.

**Table 1:** Characteristics of the infants.

Serial number	Sex	GA (W)	BW (g)	Delivery mode	IVF	Intrauterine infection	Weight of 40 W CA	Dex dose	Apgar score			RDS grade	PS dose	Reintubation	Complication				
									1 min	5 min	10 min				BPD	VAP	IVH	LOS	NEC
Patient 1	M	27	1180	VD	N	N	2300	1	–	–	–	3	3	N	Y	Y	Y	Y	N
Patient 2	M	28 + 3	1010	CS	N	N	2400	1	10	10	10	4	1	Y	Y	N	N	Y	N
Patient 3	F	27 + 4	1150	CS	Y	Y	3510	1	6	7	8	3	1	N	Y	Y	N	N	N
Patient 4	M	27 + 5	1120	VD	Y	Y	2680	1	5	7	–	4	1	N	Y	N	N	Y	N
Patient 5	M	26 + 5	850	VD	Y	Y	2500	1	9	9	9	3	2	N	Y	Y	N	N	Y
Patient 6	M	26 + 5	880	VD	Y	Y	2590	1	6	10	10	3	2	N	Y	Y	N	Y	N
Patient 7	M	27 + 1	730	CS	N	Y	1700	2	2	8	9	3	4	N	Y	N	Y	N	N
Patient 8	M	26 + 5	950	CS	N	Y	–	0	5	7	9	4	1	N	Y	N	N	Y	N
Patient 9	M	26 + 5	980	CS	N	Y	–	1	6	9	9	4	1	N	Y	N	Y	Y	N
Patient 10	M	28	1180	VD	N	N	2180	2	9	9	9	3	1	N	Y	N	N	N	N
Mean ± SD	—	27.1 ± 0.7	1003.0 ± 154.3	—	—	—	2482.5 ± 514.2	—	—	—	—	—	—	—	—	—	—	—	—

Abbreviations: BPD, bronchopulmonary dysplasia; BW, birth weight; CA, correct age; CS, cesarean; DEX, dexamethasone doses; GA, gestational age; hsPDA, hemodynamics patent ductus arteriosus; IVF, in vitro fertilization; IVH, intraventricular hemorrhage; LOS, late‐onset sepsis; N, no; NEC, necrotizing enterocolitis; PS, pulmonary surfactant; RDS, respiratory distress syndrome; ROP, retinopathy of prematurity; VAP, ventilation‐associated pneumonia; VD, vaginal delivery; Y, yes.

### 3.2. hUCB Characteristics

All participants received allogeneic hUCB‐MNCs infusion at postnatal 36.8 ± 14.7 days. The infants/donors pairs were 5/6 (*n* = 2), 4/6 (*n* = 3), or 3/6 (*n* = 4) partially HLA‐matched at HLA‐A, –B, and –DRB1, and 1/6 (*n* = 1) HLA mismatched. The median pre‐cryopreservation cell of the entire hUCB unit was (11.5 ± 4.93) × 10^8^ TNCs/kg (range, 8.18–24.35 × 10^8^ cells/kg). Only a portion of the post prosessed hUCB unit was used for infusion because of the low weight of infants, and the remainder was discarded. Allogeneic hUCB infusions delivered a median hUBC‐MNCs of (3.51 ± 0.93) × 10^6^ cells/kg (range, 2.35–5.55 × 10^6^ cells/kg). All hUCB‐MNCs had negative post‐thaw sterility cultures. All hUCB units exhibited CFU growth post‐thaw (Table 2).

**Table 2:** Characteristics of human umbilical cord blood‐derived mononuclear cells (hUCB‐MNCs).

Serial number	Precryopreservation				After ficoll density gradient centrifugation			HLA matches (no./total no.)	HLA mismatch			Volume (mL)	Body weight at infusion (kg)	Total MNC (×10^6^/kg)	CD34^+^ cell (×10^6^/kg)	Infused age (day)	Pathogen detection
	Volume (mL)	TNC Cells (×10^8^)	CD34^+^ (×10^6^)	CFU‐GM (×10^6^)	Volume (mL)	Total MNC concentration (×10^8^/L)	CD34^+^ (×10^6^)		Patient/donor								
									A	B	DRB1						
Patient 1	30	8.94	5	1.34	20.80	4.76	2.3	1/6	11:01	13:01 46:01	04:05 12:02	10.5	1.49	3.35	1.54	34	Negative
Patient 2	30	9.81	4.12	0.961	22.00	4.14	0.765	3/6	02:03	51:02	04:05	10.0	1.5	2.76	0.51	61	Negative
Patient 3	30	15.21	3.65	1.422	23.00	3.04	1.15	4/6	—	54:01	09:01	12.0	1.55	2.35	0.74	20	Negative
Patient 4	30	8.18	6.87	2.764	22.00	5.28	3.71	4/6	—	54	13	13.0	2.0	3.44	1.86	64	Negative
Patient 5	30	11.54	3.46	0.8308	22.00	2.46	1.2	3/6	24:02	54:01	04:05	14.0	1.07	3.22	1.12	34	Negative
Patient 6	30	9.88	4.35	0.699	20.80	3.31	2	3/6	33	44	13	13.0	1.06	4.05	1.89	34	Negative
Patient 7	30	9.75	4.19	1.969	26.00	4.18	2.93	3/6	33	58	13	10.0	1.0	4.18	2.93	35	Negative
Patient 8	30	8.54	5.55	1.827	24.00	5.50	3.07	5/6	01:01	—	—	10.0	1.0	5.55	3.07	24	Negative
Patient 9	30	9.22	3.41	1.7149	24.00	3.09	3.28	5/6	31	—	—	11	1.3	2.62	2.52	38	Negative
Patient 10	30	24.35	5.35	2.191	20.90	4.12	3.02	4/6	—	62,48	—	13.0	1.5	3.57	2.01	24	Negative
Mean ± SD	—	11.54 ± 4.93	4.60 ± 1.10	1.57 ± 0.65	22.55 ± 1.70	3.99 ± 1.01	2.34 ± 1.02	—				11.65 ± 1.53	1.35 ± 0.32	3.51 ± 0.93	1.92 ± 0.93	36.8 ± 14.77	Negative

Abbreviations: CFU‐GM, colony‐forming unit granulocyte‐macrophage; HLA, human leukocyte antigen; MNC, mononuclear cells; N/A, not applicable; TNC, total nucleated cells.

### 3.3. Outcomes

Four out of 10 enrolled infants were lost to follow‐up due to parental reasons. These four infants had no adverse events recorded during hospitalization and had no acute or chronic GVHD, respiratory or neurological complications, or infusion‐related adverse events at discharge and had no donor chimerism at day 28 postinfusion, but their safety status beyond hospital discharge could not be confirmed. The 2‐year safety conclusion was based on the six infants who completed follow‐up.

#### 3.3.1. Safety of hUCB‐MNCs Infusions

All infants tolerated hUCB‐MNC infusion well, including the 1/6 HLA‐mismatched case. No acute infusion reactions, cardiopulmonary decompensation, or acute or chronic GVHD were observed. Systematic monitoring revealed undetectable donor chimerism in all infants at 28 days postinfusion, and all corresponding test records and chimerism monitoring results are presented in Supporting Information 4: File 4. Serial CRP, liver transaminases, and bilirubin remained within normal limits. Extended monitoring confirmed no alloimmune complications. Transient grade 1 hypertension occurred in two infants shortly after infusion and resolved spontaneously, attributed to fluid volume load rather than cellular immunogenicity (Table 3 and Supporting Information file STR‐based report).

**Table 3:** Primary outcome before and after hUCB‐MNCs infusion.

Serial number	Vital sign																Transient events			Fever	Survival in hospital
	HR				SBP (mmHg)				DBP (mmHg)				SPO_2_								
	Pre	Post (h)			Pre	Post (h)			Pre	Post (h)			Pre	Post (h)							
		12	24	48		12	24	48		12	24	48		12	24	48	Transient hypertension	Increase in FiO_2_ by >30% within 6 h	Increase in MAP >5 cm H_2_O within 6 h		
Panel A																					
Patient 1	158	150	145	142	63	65	60	57	29	28	28	26	91	95	95	92	N	N	N	N	Y
Patient 2	151	142	140	141	55	58	57	56	26	30	28	27	90	96	95	92	N	N	N	N	Y
Patient 3	150	145	145	147	60	90	85	90	34	58	60	65	95	98	98	97	Mild	N	N	N	Y
Patient 4	140	141	135	130	77	90	80	119	45	60	65	78	95	100	98	95	Mild	N	N	N	Y
Patient 5	179	170	170	165	58	60	62	59	27	30	28	27	93	97	95	94	N	N	N	N	Y
Patient 6	100	120	135	160	60	60	60	58	29	28	28	26	89	93	93	92	N	N	N	N	Y
Patient 7	150	145	152	150	59	62	60	56	26	26	25	25	90	95	90	90	N	N	N	N	Y
Patient 8	145	146	147	145	51	57	55	53	26	28	27	28	65	95	90	90	N	N	N	N	Y
Patient 9	139	136	135	131	56	60	60	58	25	26	26	29	90	98	96	95	N	N	N	N	Y
Patient 10	139	142	130	131	55	59	62	59	24	28	28	28	90	95	93	90	N	N	N	N	Y
Mean ± SD	145.1 ± 19.8	143.7 ± 12.4			59.4 ± 7.0	66.1 ± 12.8			29.1 ± 6.3	34.2 ± 13.1			88.8 ± 8.6	96.2 ± 2.0							
		143.4 ± 11.6				64.1 ± 10.0				34.3 ± 14.9				94.3 ± 2.8							
		144.2 ± 12.0				66.5 ± 21.2				35.9 ± 19.0				92.7 ± 2.5							

Serial number	Grade of acute GVHD					Grade of chronic GVHD					STR‐based chimerism analysis at 28 days postinfusion	CRP (mg/mL)									
	Rash	Liver function		Diarrhea	Pulmonary GVHD	Rash	Liver function		Diarrhea	Pulmonary GVHD		Pre	Post								
		ALT	TBIL (ummol/L)				ALT	TBIL (ummol/L)					24 h	1 week	2 weeks	1 month					
Panel B																					
Patient 1	N	50	10.0	N	N	N	15	2	N	N	Not detected	1.0	1.0	1.0	1.0	1.0					
Patient 2	N	19	5	N	N	N	15	6	N	N	Not detected	1.0	1.0	1.0	1.0	1.0					
Patient 3	N	3	2	N	N	N	2	3	N	N	Not detected	1.0	1.0	1.0	1.0	1.0					
Patient 4	N	21	10	N	N	N	12	3	N	N	Not detected	1.0	1.0	1.0	1.0	1.0					
Patient 5	N	9	14	N	N	N	7	9	N	N	Not detected	7.0	8.0	1.0	1.0	1.0					
Patient 6	N	15	3	N	N	N	11	1	N	N	Not detected	1.0	8.0	5.0	1.0	1.0					
Patient 7	N	17	10	N	N	N	16	5	N	N	Not detected	1.0	1.0	1.0	1.0	1.0					
Patient 8	N	4	5.0	N	N	N	27	3.0	N	N	Not detected	4.0	5.0	5.0	1.0	1.0					
Patient 9	N	15	8.0	N	N	N	10	6.0	N	N	Not detected	11.0	6.0	4.0	1.0	1.0					
Patient 10	N	50	10.0	N	N	N	84	8.0	N	N	Not detected	1.0	1.0	1.0	1.0	1.0					
Mean ± SD	—	20.3 ± 16.8	7.7 ± 3.8	—	—	—	19.9 ± 23.4	4.6 ± 2.6	—	—	—	2.9 ± 3.5	3.3 ± 3.1	2.1 ± 1.8	1.0 ± 0.0	1.0 ± 0.0					

*Note*: STR‐based chimerism analysis, short tandem repeat‐based chimerism analysis. Post, after hUCB‐MNCs infusion; Pre, before hUCB‐MNCs infusion; SPO_2_, pulse oxygen saturation.

Abbreviations: CTCAE, common terminology criteria for adverse events; DBP, diastolic pressure; DSA, donor‐specific antibodies; FiO_2_, fraction of inspiration O_2_; h, hour; HR, heart rate; MAP, mean airway pressure; N, no; SBP, systolic pressure; Y, yes.

Six infants completed the 24‐month follow‐up. No SAEs or GVHD were detected. One infant was rehospitalized for community‐acquired pneumonia at 12 months, and one infant was diagnosed with cerebral palsy; neither event was related to hUCB‐MNCs infusion (Table 4). All clinical events were reported in accordance with CTCAE v5.0, and a complete AE/SAE line list is provided in Supporting Information 5: Table S1.

**Table 4:** Follow‐up outcome.

Serial number	NDS score				Rehospitalization because of pneumonia		Home oxygen therapy		Blindness		Deafness		AEs		GVHD	
	Visit 1		Visit 2		Visit 1	Visit 2	Visit 1	Visit 2	Visit 1	Visit 2	Visit 1	Visit 2	Visit 1	Visit 2	Visit 1	Visit 2
	Score	Grade	Score	Grade												
Patient 1	104	Average	102	Average	N	N	N	N	N	N	N	N	N	N	N	N
Patient 2	102	Average	105.5	Average	Y	N	N	N	N	N	N	N	N	N	N	N
Patient 3	110	Average	118	Good	N	N	N	N	N	N	N	N	N	N	N	N
Patient 4	98	Average	103.5	Average	N	N	N	N	N	N	N	N	N	N	N	N
Patient 5	118	Good	120	Good	N	N	N	N	N	N	N	N	N	N	N	N
Patient 6	Cerebral palsy				N	N	N	N	N	N	N	N	N	N	N	N
Patient 7	Parents request to therapy interruption due to personal reasons and arrange for discharge. Subsequently lost contact and was unable to follow up on the infants															
Patient 8	Parents request to therapy interruption due to personal reasons and arrange for discharge. Subsequently lost contact and was unable to follow up on the infants															
Patient 9	Parents request to therapy interruption due to personal reasons and arrange for discharge. Subsequently lost contact and was unable to follow up on the infants															
Patient 10	Parents request to therapy interruption due to personal reasons and arrange for discharge. Subsequently lost contact and was unable to follow up on the infants															

Abbreviation: NDS, neuropsychological development sale score.

#### 3.3.2. Secondary Outcome

After hUCB‐MNCs infusion, five infants weaned MV to CPAP and NC flow successfully within 1 weeks, one infant needed reintubation because of hsPDA, one infant with MV received FiO_2_ = 0.3 (decreased FiO_2_ ranging from 60% to 30%) at discharge, and three infants showed no significant improvement in respiratory support following infusion (Table 3).

#### 3.3.3. Other Outcomes

**Figure 2:**
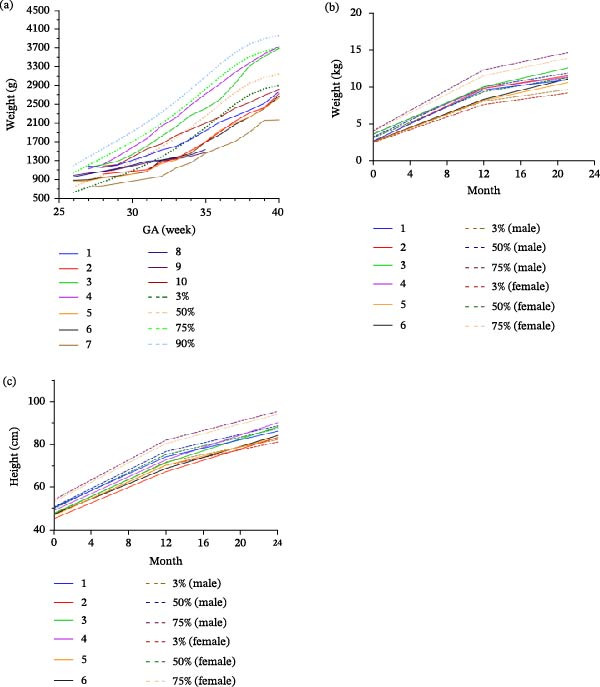
Body weight and height during hospitalization and 24‐month follow‐up. (a) Body weight during hospitalization before CA 40 weeks. (b) Body weight during 24‐month follow‐up. (c). Height during 24‐month follow‐up.

The duration of hospitalization ranged from 44 to 129 days (Table 5), although treatment was discontinued in two infants at a postmenstrual age of 33–34 weeks due to family‐related reasons. Body weight during hospitalization was 50%–75% of that in infants of the same GA in two infants and below 30% in the remaining eight infants (Figure 2). In the first 24 h of hUBC‐MNCs infusion, the pH value improved in all infants as compared with the baseline value (Table 6). No significant difference in WBC, PLT, Hb, and blood gas (PO_2_ and PCO_2_) after 24 h, a week, and 4 weeks of hUBC‐MNCs infusion (Table 5). None of the infants developed abnormal clinical features of central nervous system disorders such as convulsions, apnea, or dysphagia. No LOS, VAP, Klebsiella and *Candida albicans* infections, or *Pseudomonas aeruginosa* pneumonia were observed in any infant after hUCB‐MNCs infusion. No patient developed NEC after hUCB‐MNCs infusion. Four infants developed IVH, which did not further progress after hUCB‐MNCs infusion.

**Table 5:** Secondary outcomes before and after hUCB‐MNCs infusion.

Serial number	Ventilator parameters												Duration of hospitalization		
	Pattern		FiO_2_ (%)		PIP		PEEP		Duration of MV/CPAP		Duration of NC				
	Pre	Post	Pre	Post	Pre	Post	Pre	Post	Pre	Post	Pre	Post	Pre	Post	Total
Patient 1	SIMV	SIMV/CPAP	30	23	22	12	6	6	34	1/17	0	9	34	37	71
Patient 2	SIMV	CPAP	30	21	22	–	6	6	61	0/3	0	9	61	22	83
Patient 3	SIMV	SIMV/CPAP	30	23	22	13	5	5	20	1/42	0	20	20	57	77
Patient 4	SIMV	SIMV/CPAP	30	25	23	13	6	6	64	3/10	0	22	64	55	119
Patient 5	SIMV	SIMV/CPAP	35	28	22	14	6	6	34	1/32	0	18	34	65	99
Patient 6	SIMV	SIMV/CPAP	30	25	22	17	5	6	34	11/31	0	19	34	95	129
Patient 7	SIMV	SIMV	47	33	24	19	5	4	35	63/0	0	0	35	63	98
Patient 8	HFO	HFO	60	30	–	–	15	15	24	20/0	0	0	24	20	44
Patient 9	SIMV	SIMV	31	28	24	18	5	5	38	6/0	0	0	38	6	44
Patient 10	SIMV	SIMV	100	95	24	24	5	5	24	73/0	0	0	24	73	97
Mean ± SD	—	—	42.3 ± 22.6	33.1 ± 22.0	22.7 ± 1.0	16.3 ± 4.0	6.4 ± 3.1	6.4 ± 3.1	36.8 ± 14.8	17.9 ± 27.2 13.5 ± 16.1	—	—	36.8 ± 14.8	49.3 ± 27.4	86.1 ± 28.3

*Note*: CPAP, nasal continuous positive airway pressure; NC, nasal cannula flow; Post, postinfusion; Pre, preinfusion.

Abbreviations: MV, mechanical ventilation; NIPPV, noninvasive positive pressure ventilation.

**Table 6:** Laboratory investigations previous and post infusion.

Serial number	Complete blood count											
	HB (g/L)				WBC (×10^9^/L)				PLT (×10^9^/L)			
	Pre	Post			Pre	Post			Pre	Post		
		24 h	1 week	4 weeks		24 h	1 week	2–4 weeks		24 h	1 week	2–4 weeks
Patient 1	129	129	148	142	14.98	13.29	17.61	14.37	205	245	399	369
Patient 2	163	112	122	123	9.5	8.23	6.38	4.12	399	385	315	393
Patient 3	139	119	144	138	15.16	14.02	9.47	7.52	359	287	298	303
Patient 4	115	123	109	126	6.43	13.59	11.84	13.86	386	412	348	495
Patient 5	159	123	113	129	12.27	8.23	7.37	8.16	368	286	224	221
Patient 6	145	142	116	123	7.67	10.76	13.50	14.36	206	239	286	326
Patient 7	146	129	99	157	12.07	15.62	7.87	12.72	125	375	387	212
Patient 8	102	97	113	100	51.71	38.53	25.32	13.86	415	461	332	256
Patient 9	102	135	118	Treatment interruption	15.98	16.52	19.28	Treatment interruption	100	63	68	Treatment interruption
Patient 10	119	119	114	137	18.40	16.36	10.29	7.95	404	422	315	335
M ± SD	131.9 ± 22.04	122.8 ± 12.5			16.42 ± 12.97	15.52 ± 8.64			296.70 ± 123.70	317.50 ± 118.52		
		119.6 ± 15.2			—	12.89 ± 6.11			—	297.20 ± 94.79		
		130.56 ± 15.8			—	10.77 ± 3.85			—	323.33 ± 89.70		
F	1.471				1.703				0.163			
P	0.256				0.191				0.920			
−2LL	304.457				247.455				426.937			
AIC	310.457				253.455				432.937			

Bloodgas												
	PH				PO_2_ (mmHg)				PCO_2_ (mmHg)			
	Pre	Post			Pre	Post			Pre	Post		
		24 h	1 week	4 weeks		24 h	1 week	2–4 weeks		24 h	1 week	2–4 weeks

Patient 1	7.38	7.39	7.47	7.39	105	63	65	51	51	46	37	39
Patient 2	–	–	7.67	7.47	–	–	145	125	–	–	19	34
Patient 3	7.32	7.38	7.36	7.40	101	192	84	121	55	46	48	58
Patient 4	7.37	7.38	–	–	117	51	–	–	35	55	–	—
Patient 5	7.39	7.51	7.40	7.34	84	64	112	35	51	24	38	58
Patient 6	7.29	7.37	7.37	7.49	35	63	51	67	57	51	47	36
Patient 7	7.43	7.39	7.37	7.38	83	62	59	60	53	50	48	46
Patient 8	7.12	7.40	7.42	7.34	29	56	124	69	90	30	37	42
Patient 9	7.38	7.41	7.39	Treatment interruption	67	66	61	Treatment interruption	48	37	53	Treatment interruption
Patient 10	7.339	7.40	7.35	7.43	32	42.5	52	47	67	51	44.9	44.8
M ± SD	7.34 ± 0.09	7.40 ± 0.04			72.56 ± 33.65	73.28 ± 45.16			56.33 ± 15.17	43.33 ± 10.63		
		7.42 ± 0.10			—	83.67 ± 34.88			—	41.32 ± 10.11		
		7.41 ± 0.06			—	71.83 ± 33.43			—	44.73 ± 9.15		
F	2.337				—	0.271			—	2.747		
P	0.107				—	0.845			—	0.067		
−2LL	−63.027				—	316.918			—	247.357		
AIC	−57.027				—	322.918			—	253.357		

*Note:* Linear mixed‐effects models were used to analyze repeated‐measures outcomes at four time points. The compound symmetry (CS) covariance structure was selected based on the minimum Akaike information criterion (AIC) and the model fit was good. Type III tests of fixed effects showed no significant main effect of time (*F* = 1.471, *p* = 0.245). Using the fourth time point as the reference, pairwise comparisons revealed no significant differences between the first, second, or third time points and the reference (*p* = 0.819, 0.296, and 0.140, respectively; all *p* > 0.05). PCO_2_, partial pressure of carbon dioxide; PO_2_, partial pressure of oxygen; Post, postinfusion; Pre, preinfusion.

Abbreviations: HB, hemoglobin; HCT, hematocrit; PH, power of hydrogen; PLT, platelet count; WBC, white blood cell count.

### 3.4. Biological Outcomes

**Figure 3:**
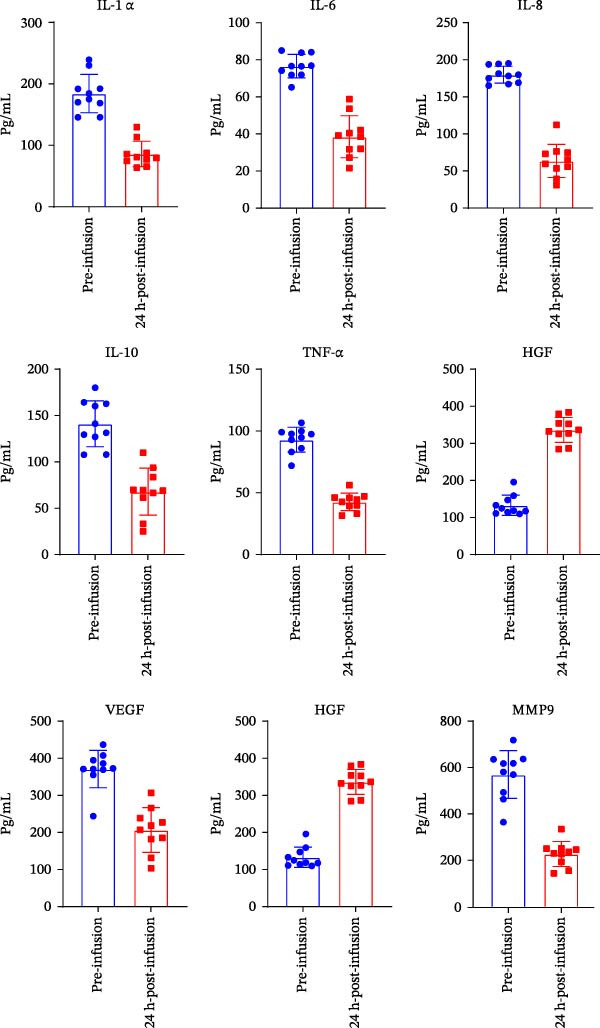
Expression profiles of inflammatory factors and growth factors in peripheral blood at baseline and after hUCB‐MNCs infusion.  ^∗^ *p* < 0.05,  ^∗∗^ *p* < 0.01,  ^∗∗∗^ *p* < 0.001, difference between baseline and hUCB‐MNCs infusion in peripheral blood. Peripheral blood was collected from the 10 extremely preterm infants before infusion and 24 h after infusion. The protein expression levels of cytokines and growth factors were determined by ELISA. IL‐10, interleukin‐10; IL‐1a, interleukin‐1a; IL‐6, interleukin‐6; IL‐8, interleukin‐8; MMP‐9, matrix metalloproteinase‐9; TGF‐β1, transforming growth factor‐β1; TNF‐α, tumor necrosis factor α; VEGF, vascular endothelial growth factor.

Serum levels of IL‐1α, IL‐6, IL‐8, IL‐10, VEGF, and MMP‐9 were significantly decreased, while HGF was significantly increased 24 h after hUCB‐MNCs infusion (all *p* < 0.05, paired *t*‐test). IFN‐α and TGF‐β1 were also significantly reduced (both *p*  < 0.05, Wilcoxon signed‐rank test) (Figure 3 and Supporting Information 6: Table S3).

### 3.5. 24‐Month Follow‐Up Outcomes

Six of the 10 infants survived to the last follow‐up visit, and the other four infants were lost to follow‐up. No infant who received hUCB‐MNCs infusion needed home oxygen therapy for long‐term respiratory complications at each visit. The other five surviving infants who received hUCB‐MNCs infusion were not diagnosed with cerebral palsy, blindness, and deafness. Brain MRI performed at 40 weeks and 9 months of CA is shown in Supporting Information 7: File 2, and neurodevelopmental assessment demonstrated an NDS score >70 in these five infants (Table 4). All six surviving infants gained weight and height comparable to age‐matched peers at visit 1 and visit 2 (Figure 2).

## 4. Discussion

We report the results of an open‐label pilot study investigating the safety of intravenous infusions of allogeneic, partially HLA‐matched or HLA‐mismatched, plasma‐, and RBC‐reduced hUCB‐MNCs in preterm infants. Infusions were administered without prior conditioning or immunosuppression and were overall well tolerated. Two infants developed transient hypertension attributed to the volume load shortly after infusion, which resolved spontaneously without any intervention. No therapy‐related SAEs or GVHD were observed either acutely or during the 2‐year follow‐up period. Notably, plasma inflammatory cytokine levels showed exploratory alterations following the infusion compared with baseline. One infant was diagnosed with cerebral palsy at the first follow‐up, which was deemed unrelated to the hUCB‐MNC infusion. At the 24‐month follow‐up assessment, five of the six surviving infants exhibited no long‐term respiratory complications, developmental delay, or neurological sequelae.

The study used allogeneic hUCB‐MNCs, which differs distinctly from most recent neonatal cord blood cell therapy trials that primarily used autologous UCB‐MNCs (Table 7). Compared with prior studies, our treatment protocol differs in four key aspects, including a minimum HLA match of 1/6, no preconditioning or immunosuppression, delayed cell infusion, and a markedly lower cells. Notwithstanding these variations, our safety data remain applicable to broader preterm infant populations. Notably, the mean hUCB‐MNCs used here was (3.51 ± 0.93) × 10^6^ cells/kg, markedly lower than the conventional doses ranging from 2 × 10^7^ to 5 × 10^7^ cells/kg in previous clinical studies [29, 30], thereby minimizing immunologic risks. Notably, intravenously infused stem cells are predominantly trapped in the lungs during first‐pass passage, with only a small fraction entering the systemic circulation, which further supports the rationale of low‐dose administration without compromising safety or efficacy [31]. Second, allogeneic hUCB‐MNCs exert therapeutic effects primarily via paracrine activity and immunomodulation rather than long‐term engraftment, making the influence of HLA mismatch on immunogenicity negligible [32]. Third, hUCB‐MNCs exhibit low intrinsic immunogenicity due to limited HLA‐DR expression, which reduces the risk of alloimmune reactions and GVHD even in the absence of immunosuppressive therapy [18].

**Table 7:** Clinical trials of cord blood–derived cell therapy in neonates (2020–present).

Study	Population	Cell	Autologous/allogeneic	HLA matching	Conditioning/immunosuppression	Timing of infusion	Cell dose	Primary outcome	Safety
Tsuji 2020	Term newborns with severe HIE (*n* = 6)	UCB‐MNC	Autologous	N/A	None	12–24 h, 36–48 h, 60–72 h after birth (3 infusions)	Volume‐reduced UCB divided into three doses	All six infants survived at 30 days; 4/6 normal neurodevelopment at 18 months	No therapy–related serious adverse events
Malhotra 2020	Extremely preterm infants <28 weeks (ongoing)	UCB‐MNC	Autologous	N/A	None	Early postnatal period	Not reported	Assess safety and feasibility; prevent preterm brain injury	Pending clinical results
Cotton 2021	Term neonates with HIE (therapeutic hypothermia)	UCB‐MNC	Autologous	N/A	None	During hypothermia	1–5 × 10^7^ MNC/kg	Better Bayley‐III scores at 18–22 months vs. control	Safe and feasible; no GVHD
Dandany 2022	Preterm infants (<32 weeks)	UCB‐MNC	Autologous	N/A	None	Within 24 h after birth	2–10 × 10^7^ MNC/kg	Improved immune microenvironment; reduced inflammation	No serious adverse events
Zhuxiao 2023	Very preterm infants (BPD prevention)	UCB‐MNC	Autologous	N/A	None	Early neonatal period	Not reported	Reduced moderate/severe BPD; improved 2‐year outcomes	Safe; no obvious adverse reactions
Ren 2023	Very preterm infants	UCB‐MNC	Autologous	N/A	None	Early postnatal period	Not reported	Reduced moderate/severe BPD; higher extubation rate; anti‐inflammatory	No serious adverse events; well‐tolerated
Zhou 2025	Extremely preterm infants (CORDSaFe)	UCB‐MN	Autologous	N/A	None	Neonatal stable period	25–50 × 10^6^ cells/kg	Feasible collection and reinfusion; improved early neurodevelopment; reduced CP risk	No therapy‐related serious adverse events; well‐tolerated
Faast 2025	Extremely preterm infants <28 weeks (CORDSaFe)	UCB‐MNC	Autologous	N/A	None	Second week after birth	25–50 × 10^6^ MNC/kg	Feasible collection/infusion; no significant early neurodevelopmental benefit	Safe; no infusion‐related serious AEs
Wong 2025	Preterm infants with severe brain injury (III/IV IVH/WMI)	UCB‐TNC	Allogeneic	4/6 or 5/6 partial match	None	Within 2–3 weeks after diagnosis	50 × 10^6^ TNC/kg	Phase I: feasibility and safety	No GVHD; no serious AEs
Ren 2025	Extremely preterm infants <28 weeks (BPD prevention)	UCB‐MNC	Autologous	N/A	None	Within 24 h after birth	5 × 10^7^ MNC/kg	Survival without BPD at 36 weeks	To be announced (RCT)
Razak 2025	Preterm infants with severe brain injury (ALLO trial, ongoing)	UCB‐TNC	Allogeneic	Partially matched	None	Neonatal period	To be determined	Safety and feasibility evaluation	Pending trial completion

Abbreviations: AE, adverse event; BPD, bronchopulmonary dysplasia; CP, cerebral palsy; GVHD, graft‐versus‐host disease; HIE, hypoxic–ischemic encephalopathy; IVH, intraventricular hemorrhage; MNC, mononuclear cell; RCT, randomized controlled trial; TNC, total nucleated cell; UCB, umbilical cord blood; WMI, white matter injury.

In the study, allogeneic hUCB‐MNCs were used to treat preterm infants for whom autologous cord blood was unavailable at birth [33, 34]. Although allogeneic cell therapy theoretically carries a GVHD risk, myeloablative preconditioning was omitted in our protocol, making sustained donor cell engraftment unlikely. This is in line with previous evidence showing that donor chimerism remains transient and low‐level, typically undetectable within weeks after infusion, and is not clearly correlated with clinical efficacy [35]. Consistent with the pulmonary first‐pass effect, most infused cells are transiently retained in the lungs rather than engrafted systemically, further limiting persistent chimerism and GVHD risk [31]. Accordingly, no GVHD or other infusion‐related adverse events were noted across enrolled infants, supporting the preliminary safety of allogeneic hUCB‐MNC infusions. Although donor chimerism was not prespecified as a primary trial endpoint, chimerism analysis was performed on all study subjects. Importantly, undetectable donor chimerism via STR testing cannot rule out recipient alloimmune sensitization after cell infusion. Even with rapid clearance of infused donor cells, mismatched HLA antigens may still trigger the DSA generation. This constitutes a key study limitation, given the absence of prospective DSA testing and the inclusion of one recipient with a 1/6 HLA mismatch. Future follow‐up trials will incorporate standardized postinfusion DSA testing to systematically evaluate the risk of alloimmunization after HLA‐mismatched hUCB‐MNC administration.

Alterations in cytokines and growth factors at 24 h postinfusion warrant cautious interpretation. The small sample size (*n* = 10) and unadjusted multiple comparisons elevate the type I error risk. Significant changes in HGF, MMP‐9, and inflammatory mediators are only exploratory hypothesis‐generating results. These biomarker analyses are limited by small sample size, no control group and a single short‐term sampling point. Infants with active sepsis, hemodynamic instability, or severe infection were enrolled, and concurrent NICU treatments, including antibiotics, ventilation adjustments, steroids, and PDA therapy confound biomarker levels. We avoid causal associations between hUCB‐MNC infusion and cytokine changes and do not confirm an anti‐inflammatory effect. Larger controlled trials with preset endpoints and multiplicity adjustment are needed for validation.

This trial was a phase I safety study and was not designed to confirm efficacy. To our knowledge, this is the first study to evaluate the feasibility and safety of allogeneic hUCB‐MNC infusion as a potential intervention for preterm infants with multiple PBAC. Further studies should focus on enrolling such high‐risk preterm infants to better assess the therapeutic potential of this approach. In the present study, no further progression of LOS or NEC was observed after hUCB‐MNC infusion. During hospitalization, only two infants achieved adequate weight gain for their GA. The insufficient weight gain in the other infants was likely due to their more severe underlying clinical conditions. Fortunately, the six infants with complete follow‐up attained age‐appropriate weight and height at 12 and 24 months of corrected age. Further studies are needed to clarify the association between postnatal catch‐up growth and favorable long‐term neurodevelopmental outcomes.

### 4.1. Limitations of the Study

This phase I trial has several limitations. First, the sample size was small, and no control group was included. Second, the study population included preterm infants at a high risk of mortality, and the timing of hUCB‐MNC infusion was relatively late. Third, the loss‐to‐follow‐up rate was relatively high (4/10). However, all losses were due to personal family reasons rather than study‐related adverse events, which lessened the potential selection bias. In addition, all participants continued to receive standard medications and physical/occupational therapies during the study. Because concomitant treatments were not suspended, the observed improvements cannot be definitively attributed to hUCB‐MNC infusion. Long‐term safety conclusions require further validation in larger cohort studies, such as the registered phase II trial.

## 5. Conclusions

No major infusion‐related adverse events were observed in preterm infants with PBAC who received allogeneic hUCB‐MNC infusions. Constrained by the small sample size and participant attrition, our long‐term safety conclusions require verification in larger controlled studies.

**Nomenclature**
hCUB: Human umbilical cord blood
MNCs: Mononuclear cells
RDS: Respiratory distress syndrome
IVH: Intraventricular hemorrhage
ROP: Retinopathy of prematurity
LOS: Late‐onset sepsis
BPD: Bronchopulmonary dysplasia
NEC: Necrotizing enterocolitis
CA: Corrected age
GVHD: Graft‐versus‐host disease
NDS: Neuropsychological development sale
CPAP: Nasal continuous positive airway pressure
NC: Nasal cannula (NC) flow
PBAC: Preterm birth‐associated complications
IVF: In vitro fertilization
SAE: Serious adverse events
VAP: Ventilation‐associated pneumonia.

## Author Contributions

Conceptualization, formal analysis, resources, and writing – review and editing were performed by Zhichun Feng, Qiuping Li, Jia Chen, Yabo Mei, and Liu He. Methodology was performed by Jia Chen, Yabo Mei, Xue Du, Danhua Zhao, Zhenlan Du, Liu He, Guosheng Xing, Liping Cheng, and Yanan Gu. Validation was performed by Jia Chen, Yabo Mei, Liu He, and Zhenlan Du. Investigation was performed by Xue Du, Danhua Zhao, Zhenlan Du, Guosheng Xing, and Liping Cheng. Data curation was performed by Jia Chen, Yabo Mei, Liu He, Xue Du, and Zhenlan Du. Writing – original draft preparation was performed by Jia Chen, Yabo Mei, and Liu He. Supervision was performed by Zhichun Feng and Qiuping Li. All revisions on cell dose data as required by the reviewer were completed by Xiaofei Wei.

## Funding

This research was jointly granted by the National Key R&D Program of China (Grants 2021YFC2701700 and 2021YFC2701702).

## Disclosure

All authors have read and agreed to the published version of the manuscript. A preprint has previously been published by Chen et al. on September 15, 2020 [36].

## Ethics Statement

The study protocol and procedures were approved by the Ethics Committee of The Seventh Medical Center of PLA General Hospital, Beijing, China on July 21^st^, 2015 (Numbers 2015111 and 2020‐037).

## Consent

All participants provided a written informed consent form. The parents of the participating infants provided informed consent to publish this article, including medical data and images. Proof of consent to publish from study participants can be requested at any time and a copy of it is available to the journal.

## Conflicts of Interest

The authors declare no conflicts of interest.

## Supporting Information

Additional supporting information can be found online in the Supporting Information section.

## Supporting information


**Supporting Information 1** Table S2. Characteristics of human umbilical cord blood‐derived mononuclear cells (hUCB‐MNCs).

**Supporting Information 2** Supporting Information file 1. Flow cytometry 0846 report. CD 34^+^ accounts for 1.72%, 0.91%, 0.31%, and 0.33% of total nucleated cells. Hematopoietic stem cells (HSCs) account for 87.30%, 83.25%, 53.81%, and 83.47% of CD34^+^ cells. TNC viability rate 96.92%, 98.61%, 99.41%, and 92.75%. HSC viability rate 96.21%, 88.7%, 86.79%, and 87.44%. 0845 report: The proportion of CD14^+^ monocytes/macrophages is 56.80%; CD1C^+^/dendritic cells account for 3.15%. CD3^+^CD5^+^ mature T cells account for 13.36%. CD3^+^CD4^+^ helper T cells account for 10.43%, while CD3^+^CD8^+^ cytotoxic T cells account for 1.64%. CD3^+^CD45RA^+^ initial T cells account for 8.66%. CD3^+^CD45RO^+^ effector T/memory T cells account for 0.17%. CD3^−^CD16^+^CD56^+^ NK cells account for 8.92%.

**Supporting Information 3** Supporting Information file 3. ELISA kit test report.

**Supporting Information 4** Supporting Information file 4. STR‐based donor cell Chimerism report.

**Supporting Information 5** Table S1. Adverse events.

**Supporting Information 6** Table S3. Expression profiles of inflammatory factors and growth factors in peripheral blood at baseline and after hUCB‐MNCs infusion.

**Supporting Information 7** Supporting Information file 2. Supporting notes on pulmonary imaging and follow‐up methods.

## Data Availability

The data that support the findings of this study are available from the corresponding author upon reasonable request.
