# Supplementary material for: A Novel Cellular Therapy for Preterm Complications: Safety Profile of Allogeneic Cord Blood Mononuclear Cells in a Pilot Clinical Study
Source: Stem Cells Int. 2026 Aug 12;2026:9979914. doi: 10.1155/sci/9979914 (PMC13464116; doi:10.1155/sci/9979914)
Supplement: Supplementary file 1 [file SCI-2026-9979914-s002.pdf]

CD34-08450846

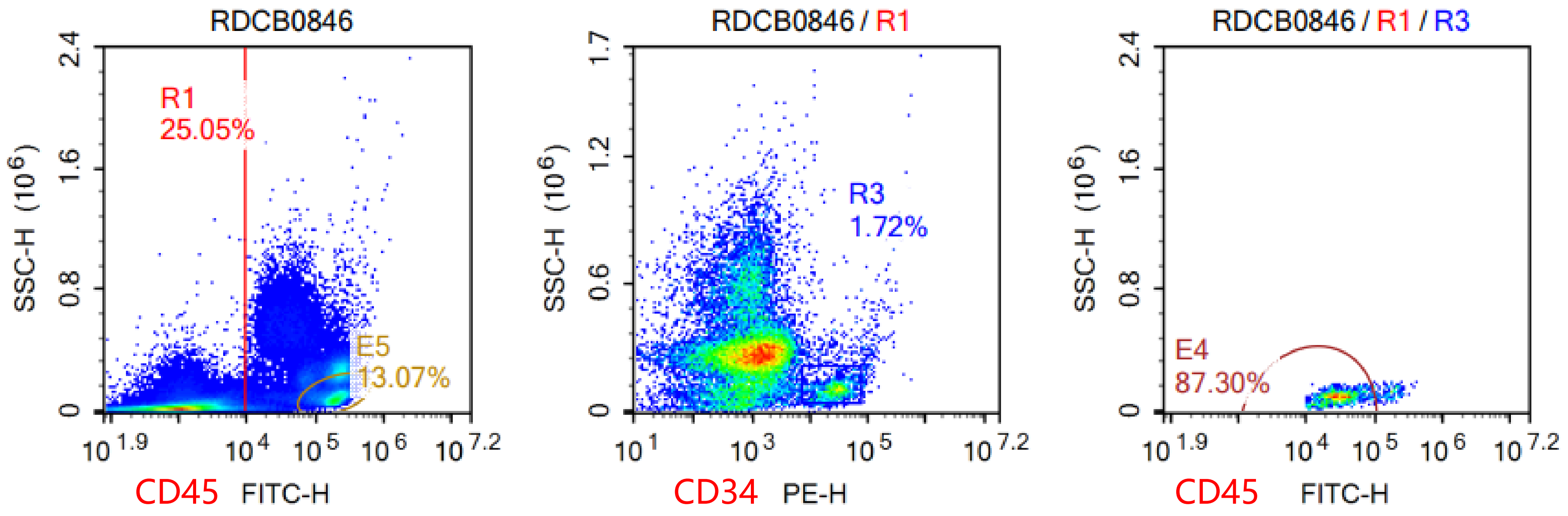

CD 34+accounts for 1.72% of total nucleated cells

Hematopoietic stem cells (HSC) account for 87.30% of CD34+cells

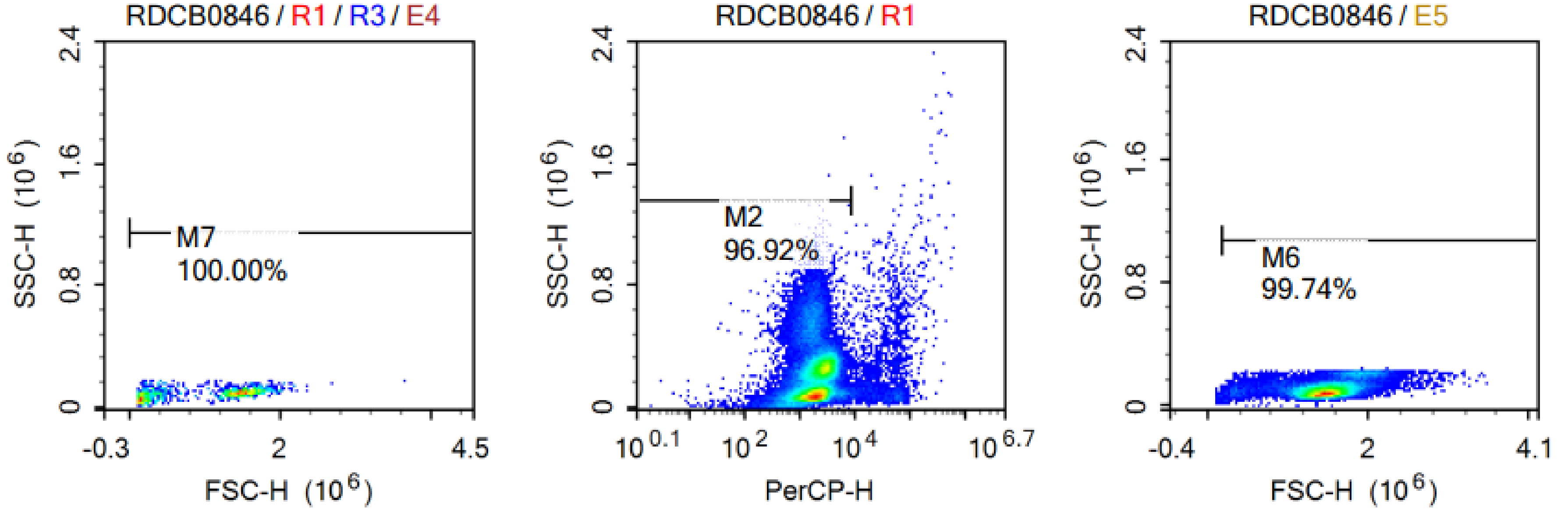

TNC Viability Rate 96.92%

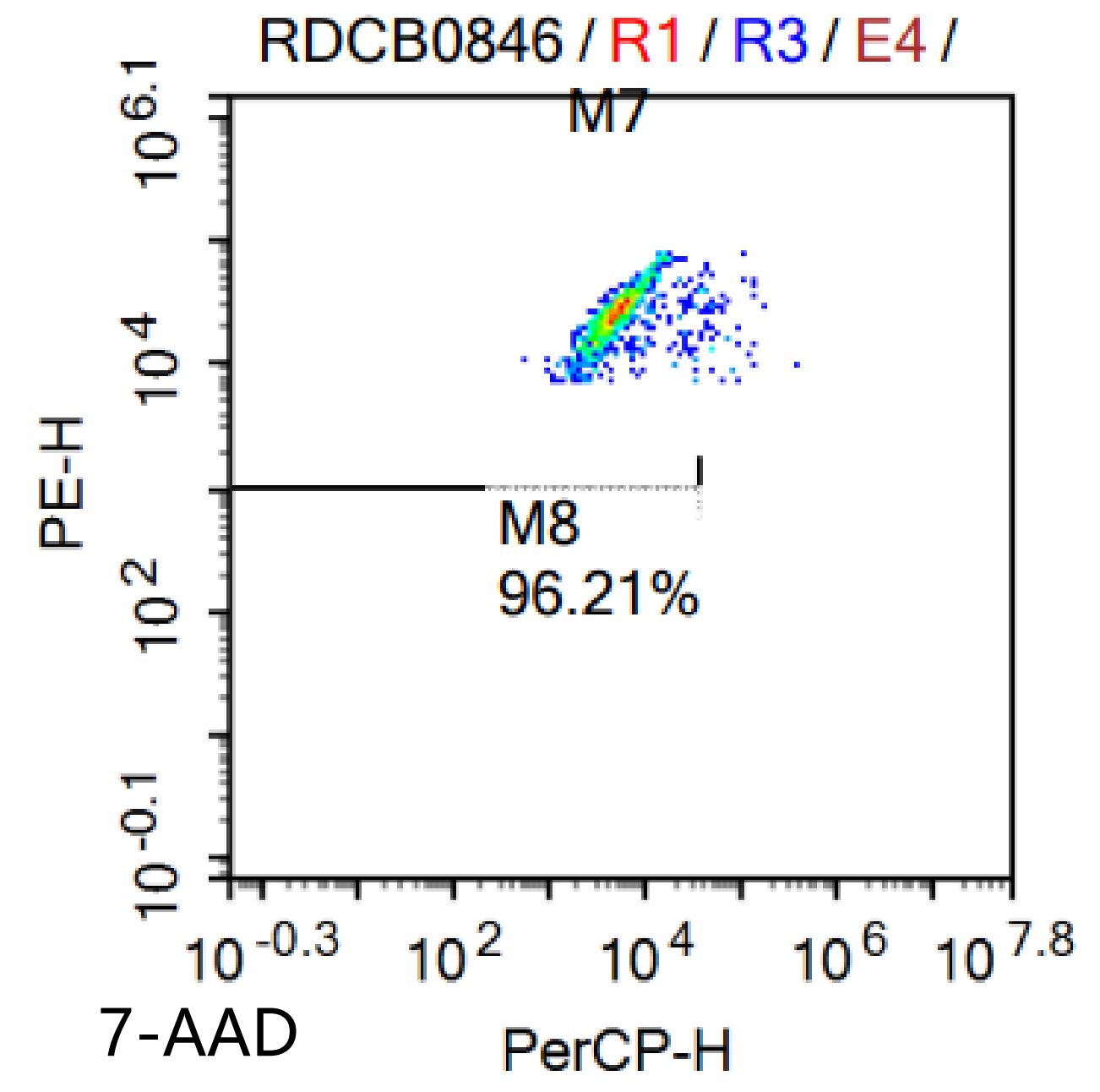

HSC Viability Rate 96.21%

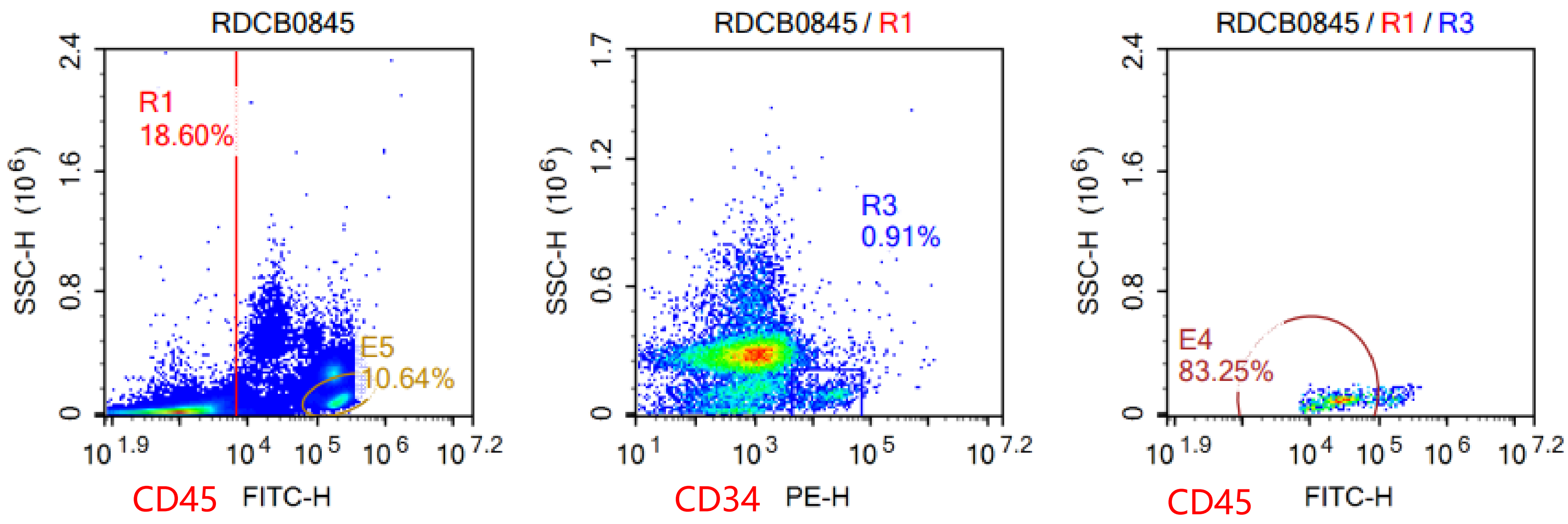

CD 34⁺ accounts for 0.91% of TNCs, while HSC make up 83.25% of CD34⁺ cells

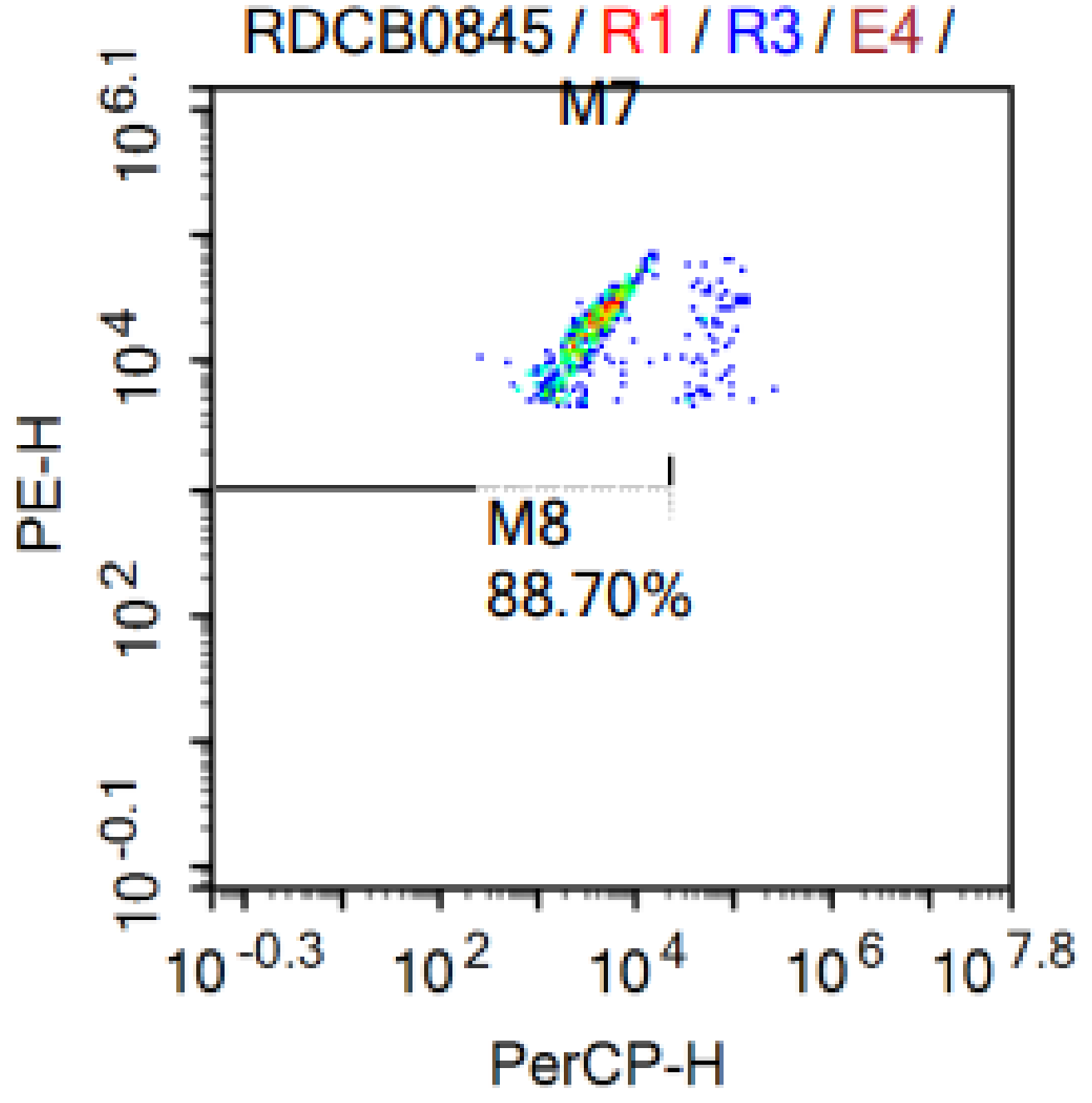

HSC Viability Rate 88.70%

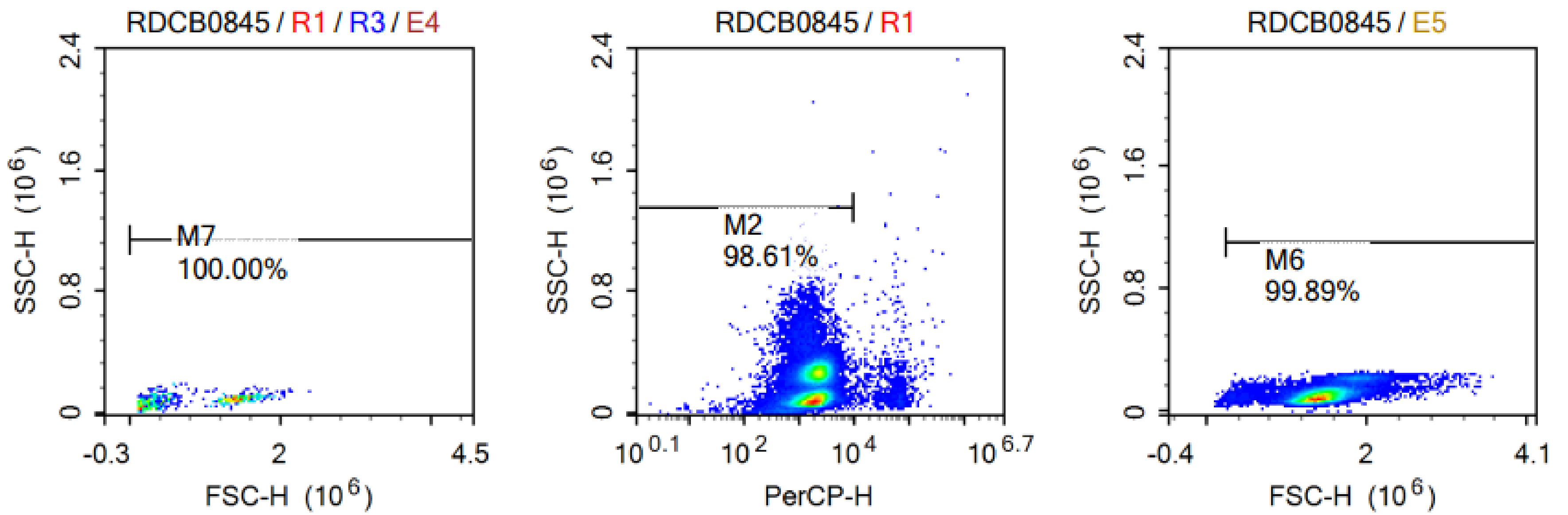

TNC Viability Rate 98.61%

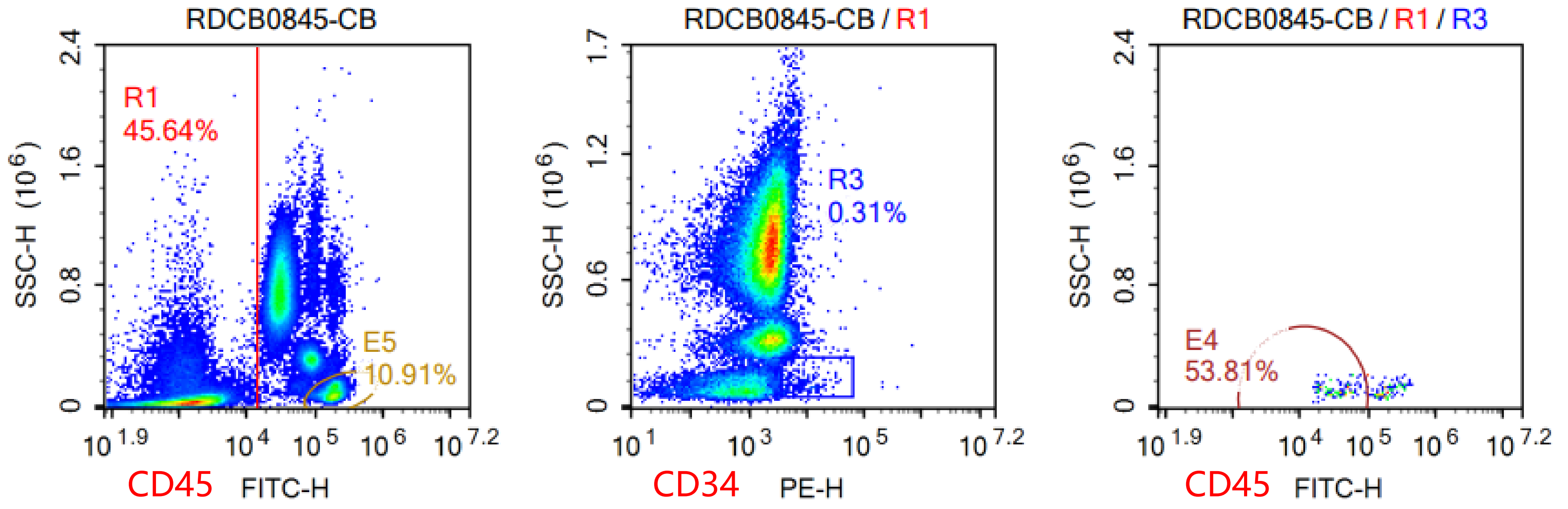

CD 34$^+$accounts for 0.31% of TNC,HSC account for 53.81% of CD34$^+$cells

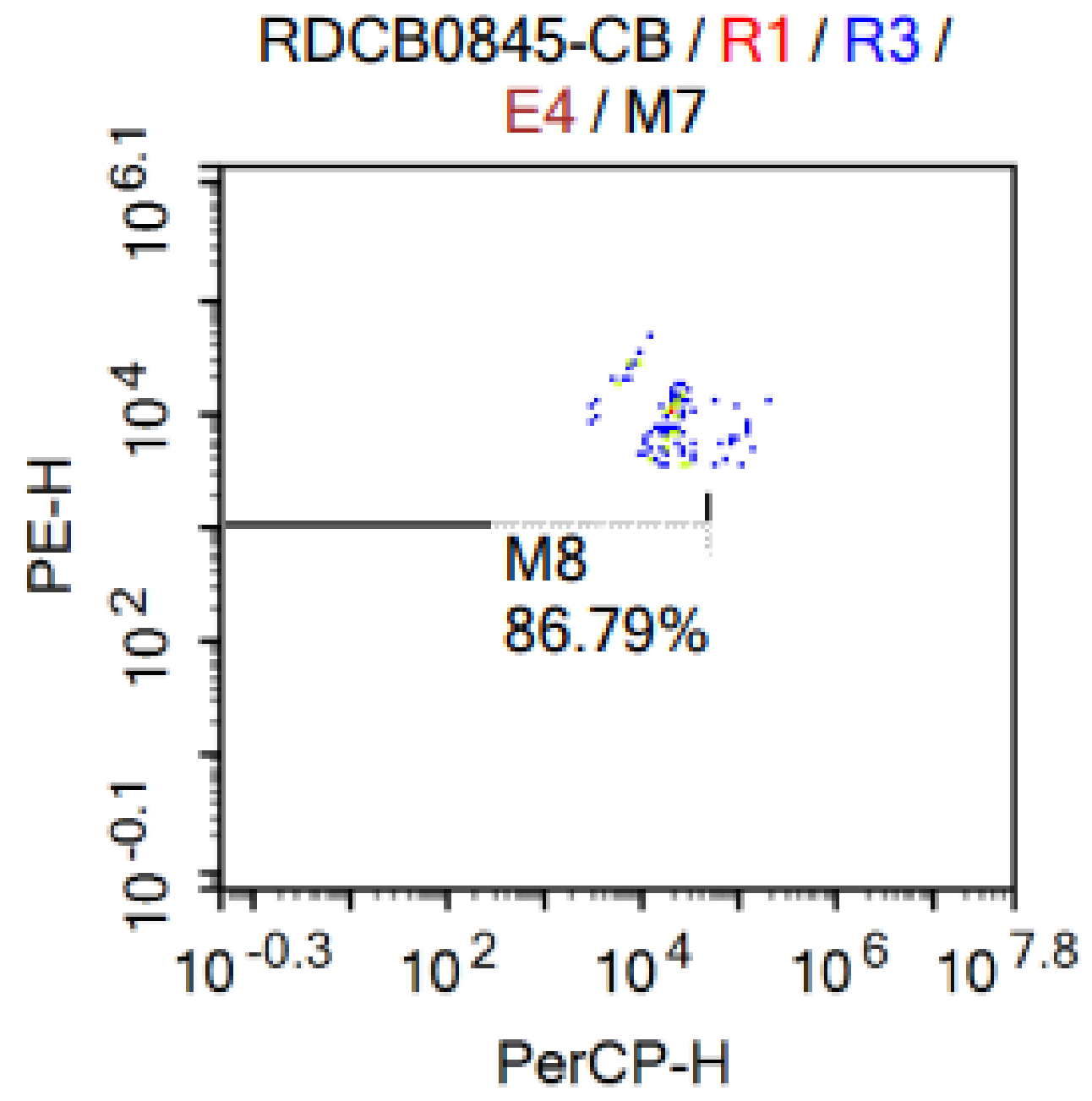

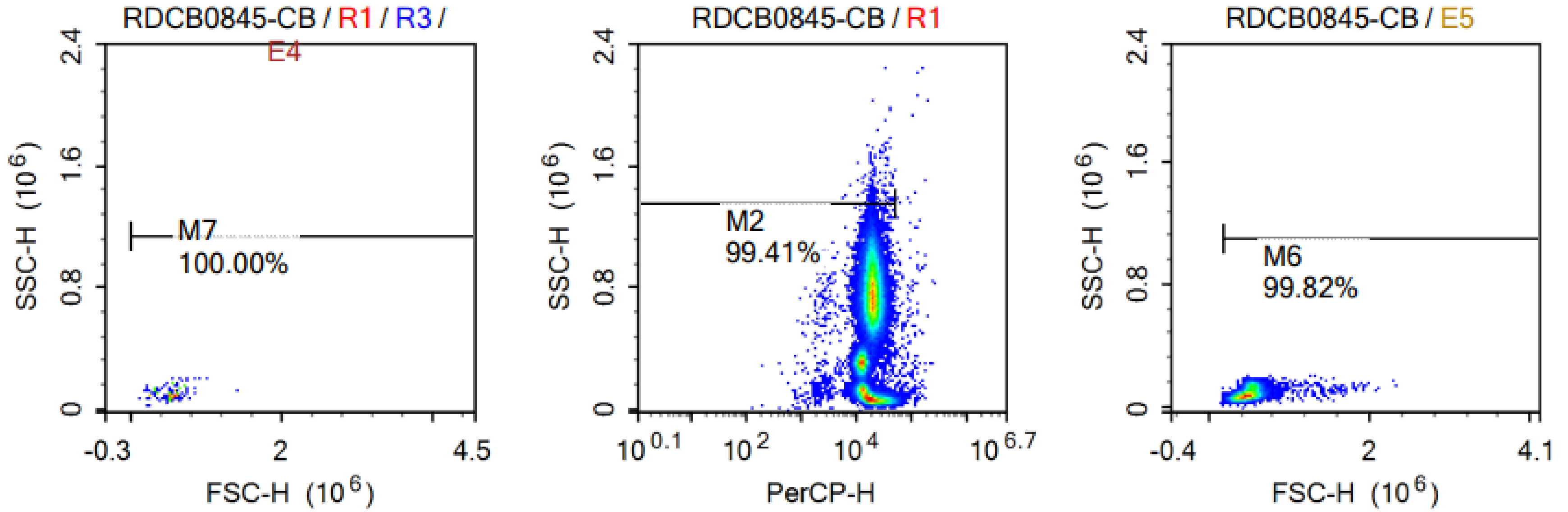

HSC Viability Rate 86.79%

TNC Viability Rate99.41%

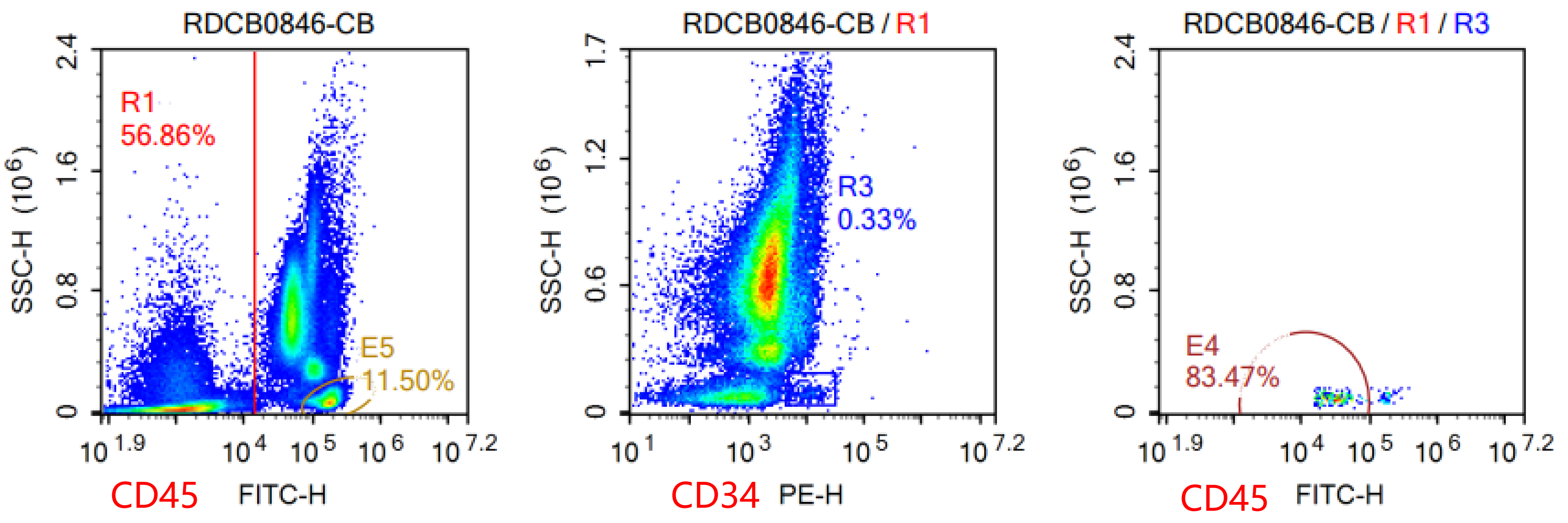

CD 34+accounts for 0.33% of TNC，HSC account for 83.47% of CD34+cells

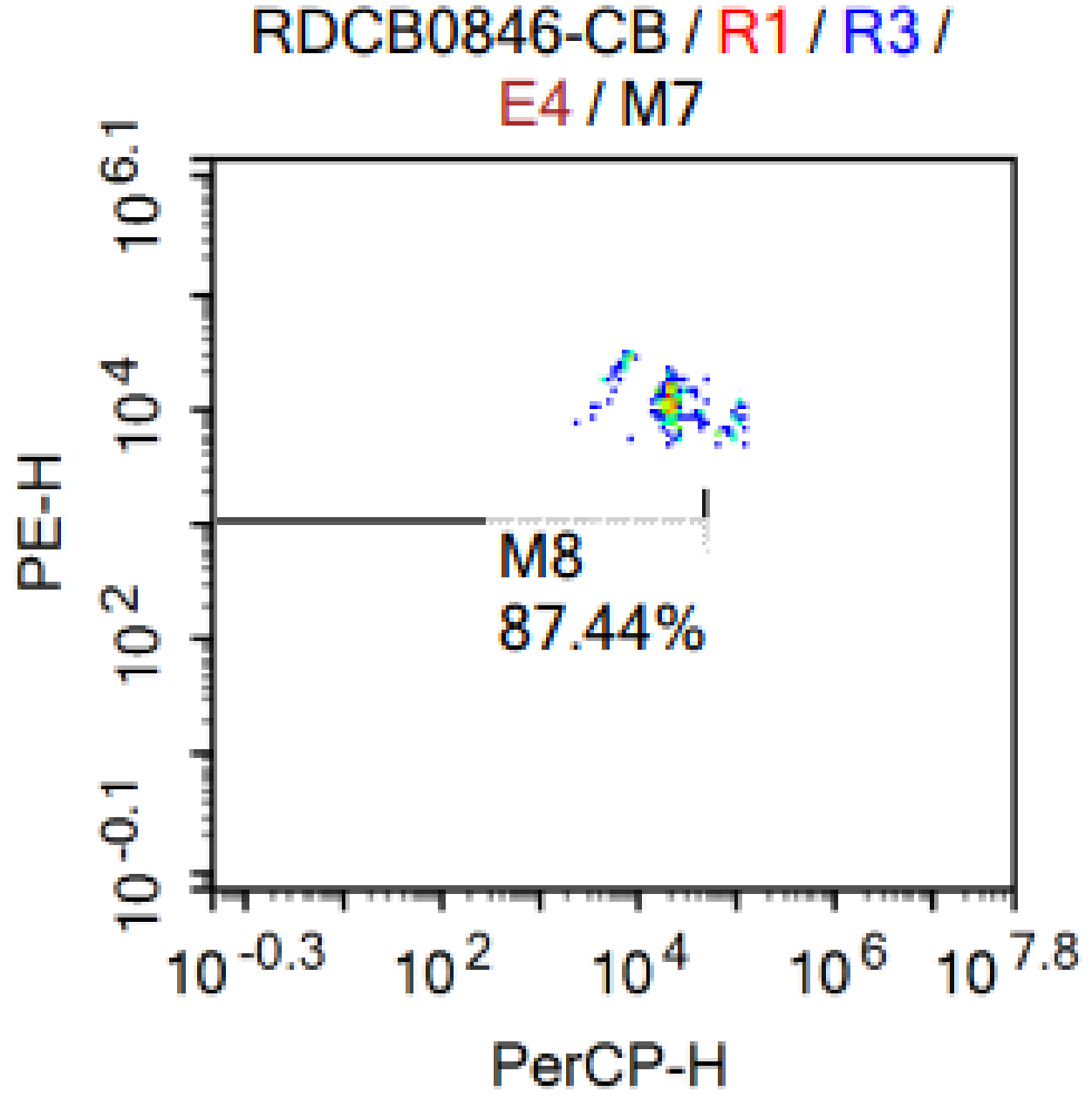

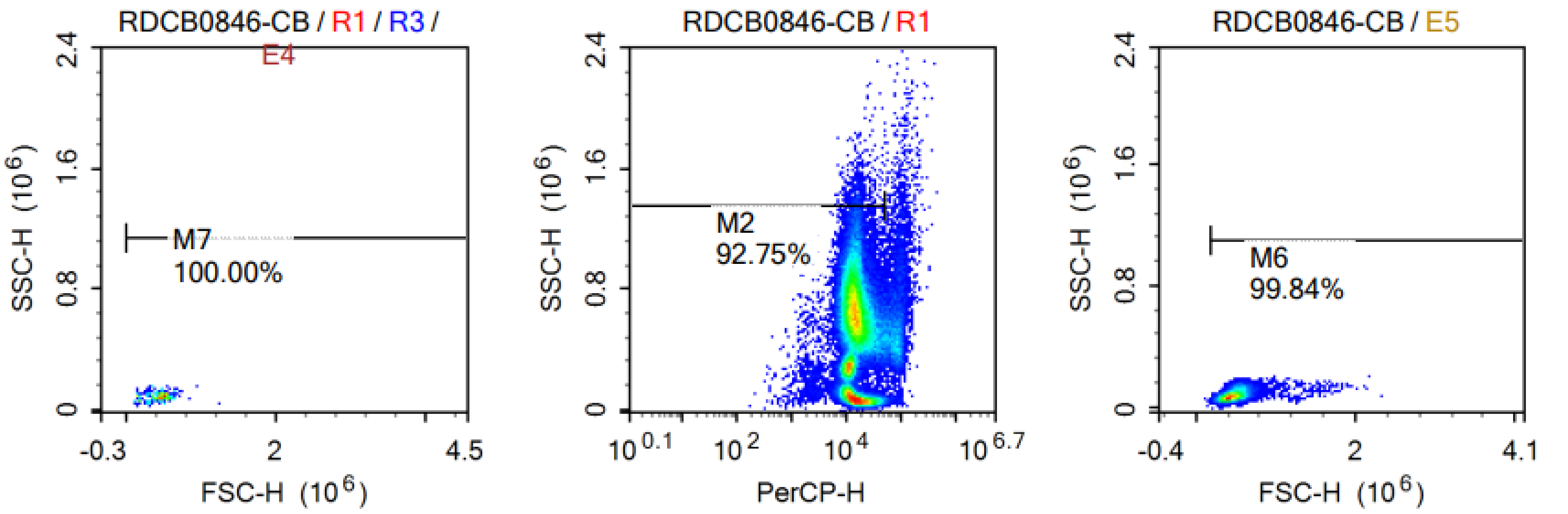

HSC Viability Rate 87.44%

TNC Viability Rate 92.75%

0845报告

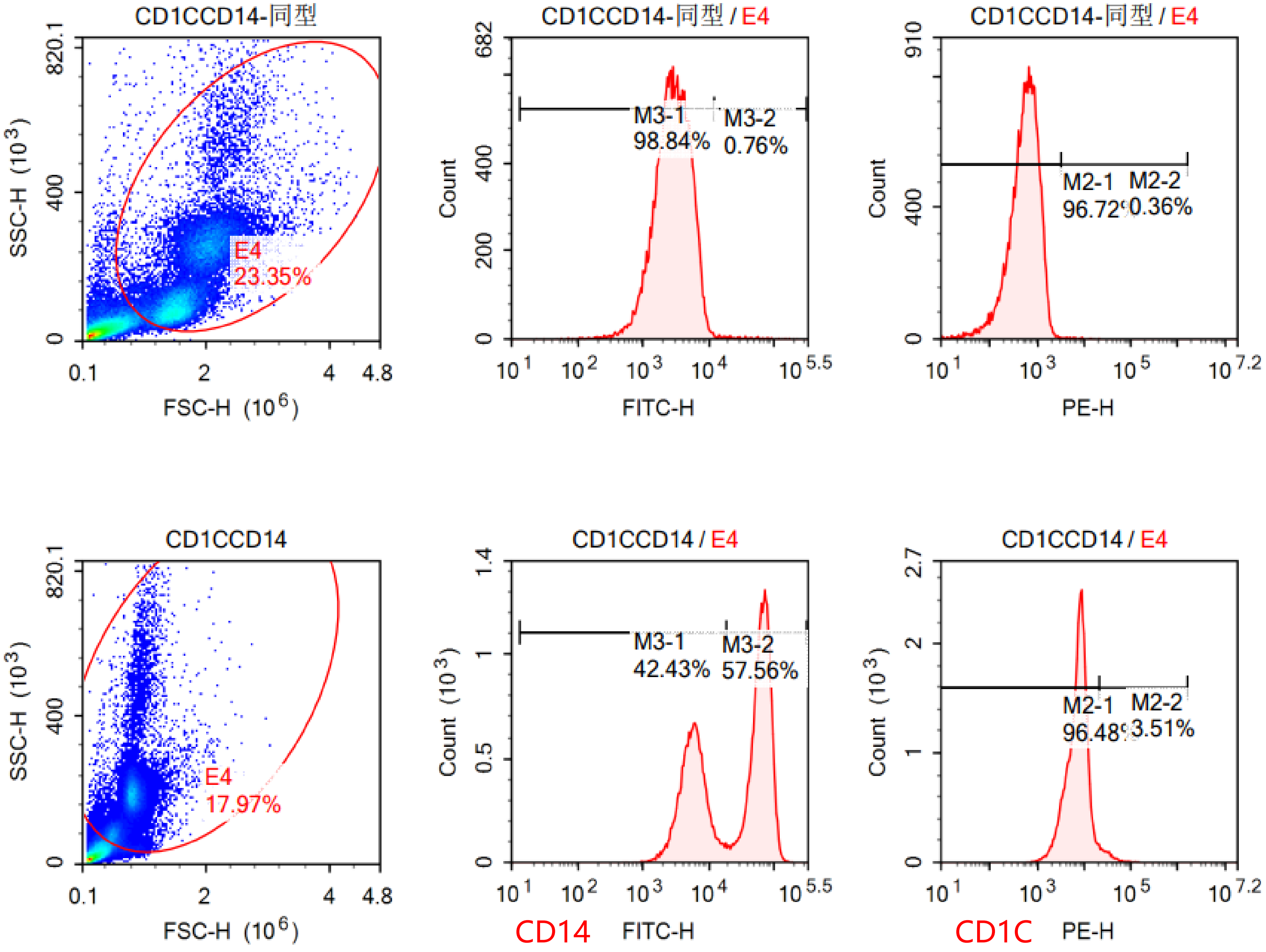

The proportion of CD14+monocytes/macrophages is 56.80%; CD1C+/dendritic cells account for 3.15%

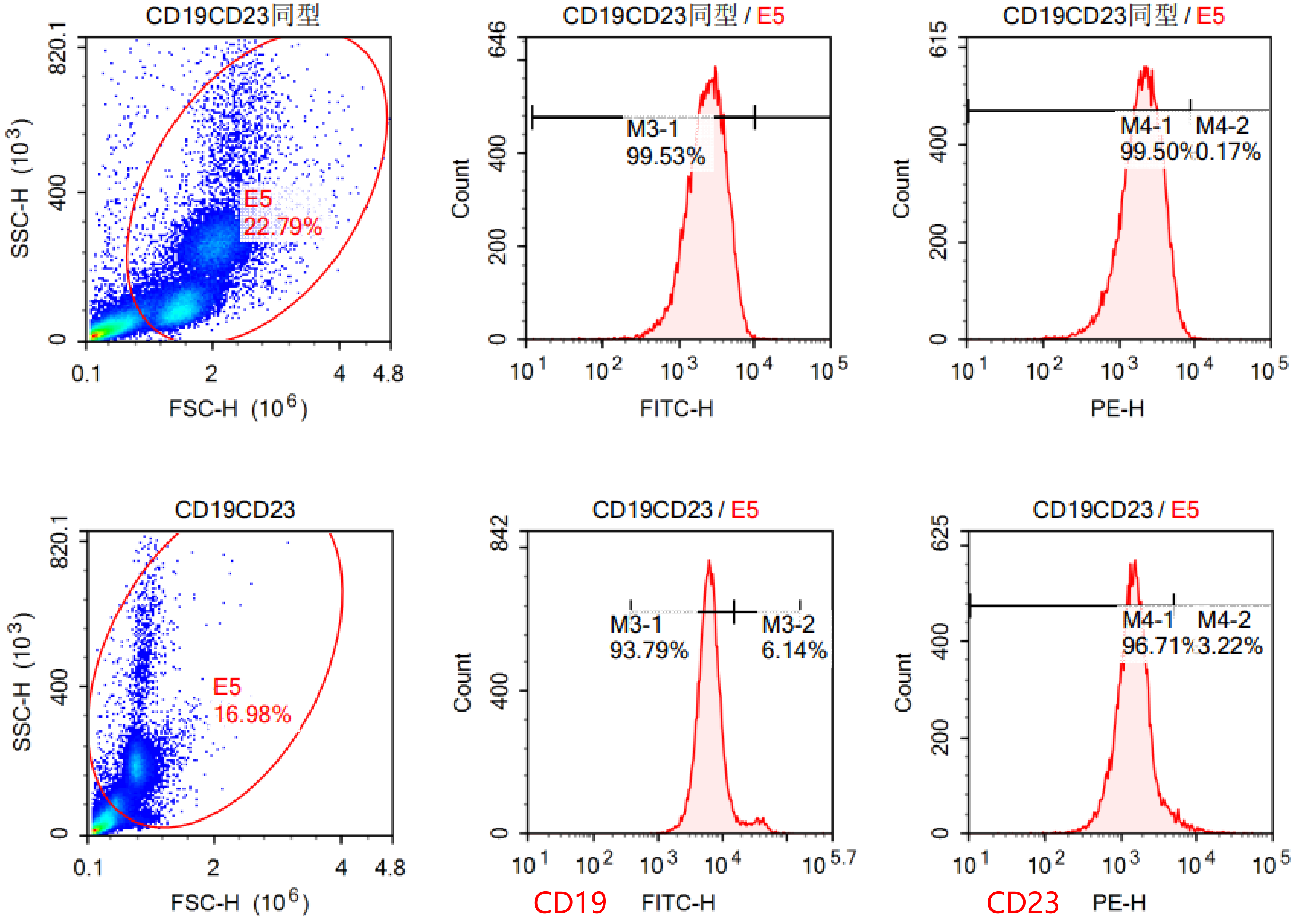

The proportion of CD19+B cells is 5.67%; CD23+B lymphocytes and follicular dendritic cells account for 3.05%

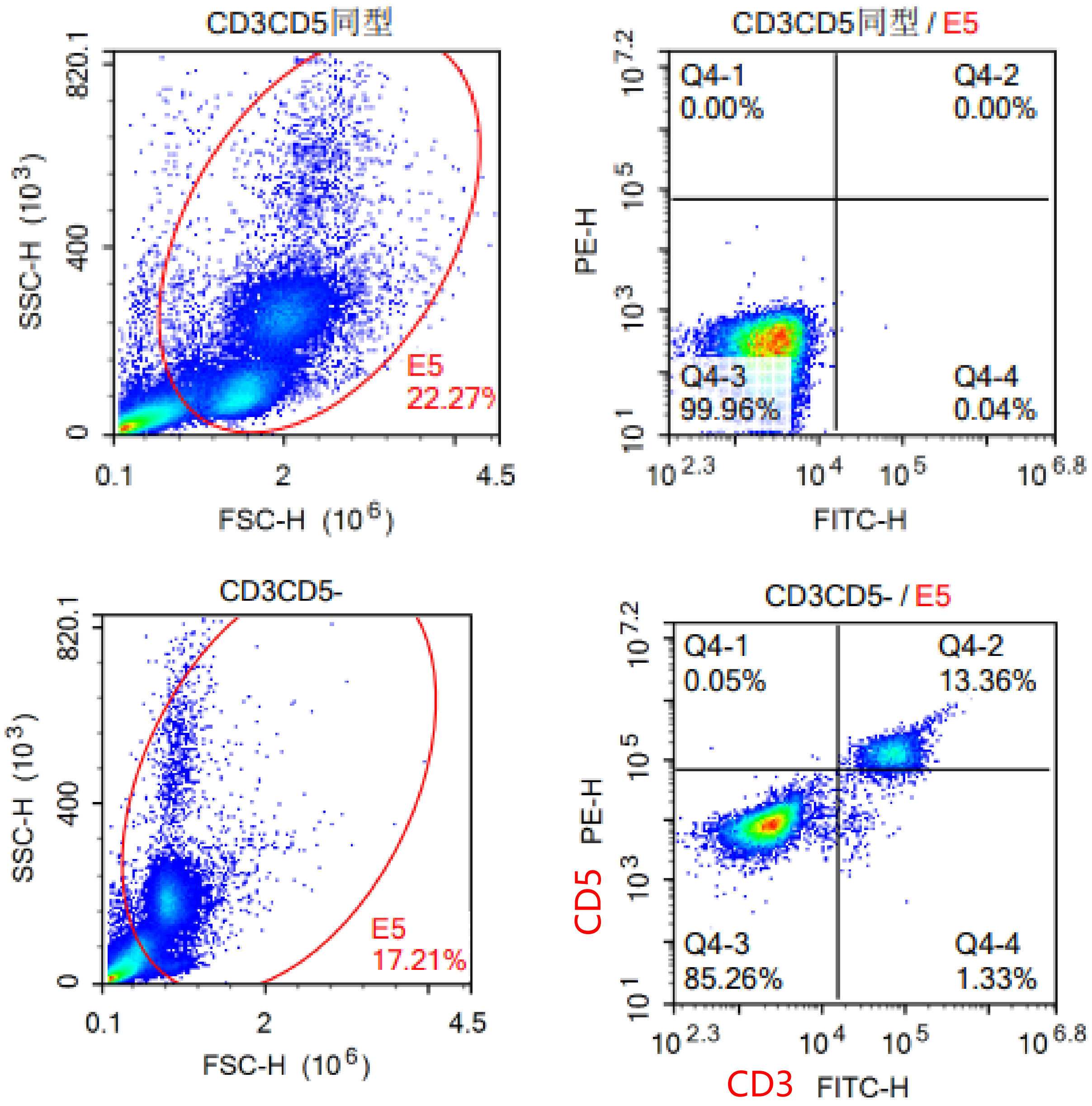

CD3+CD5+mature T cells account for 13.36%

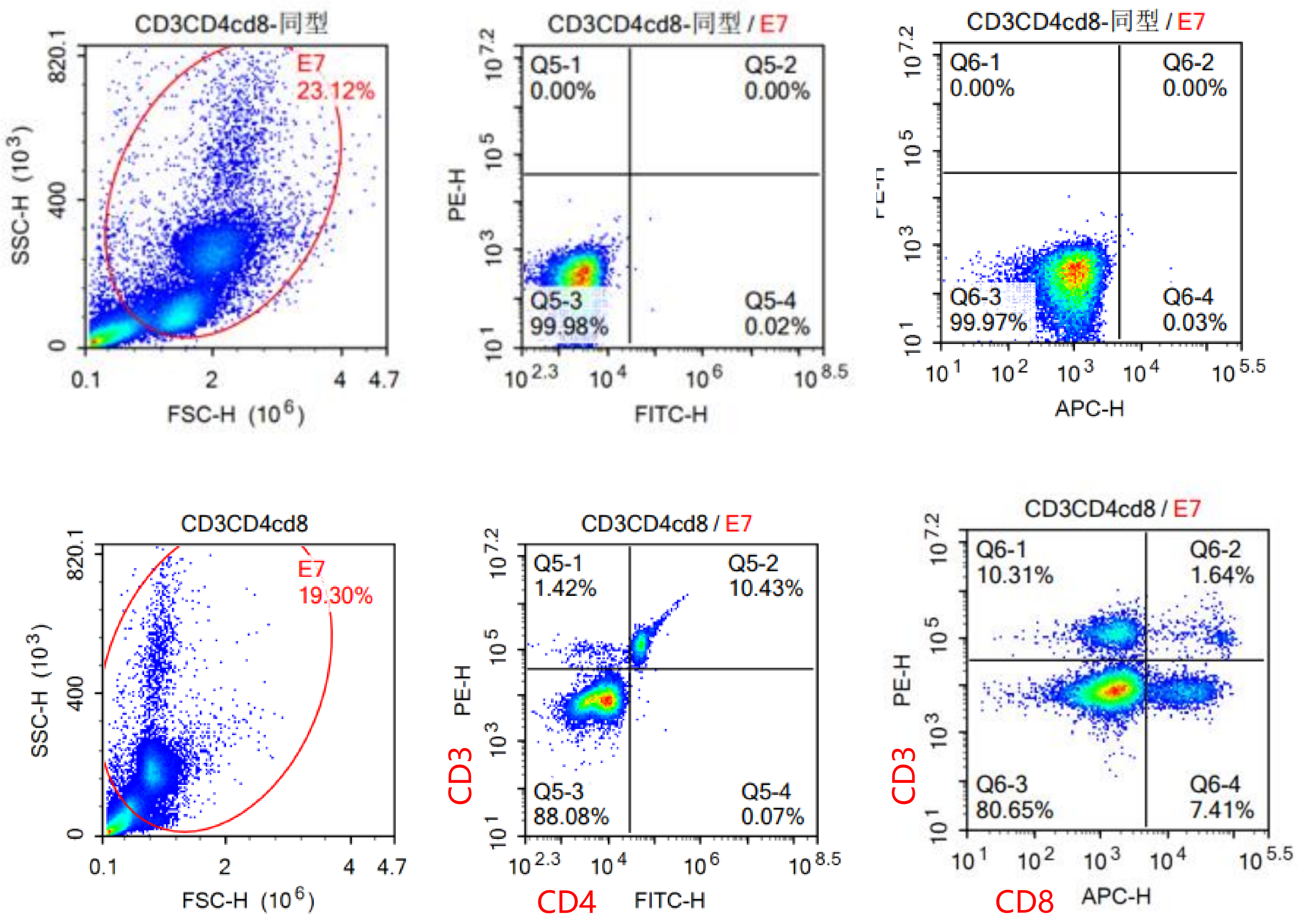

CD3+CD4+helper T cells account for 10.43%, while CD3+CD8+cytotoxic T cells account for 1.64%

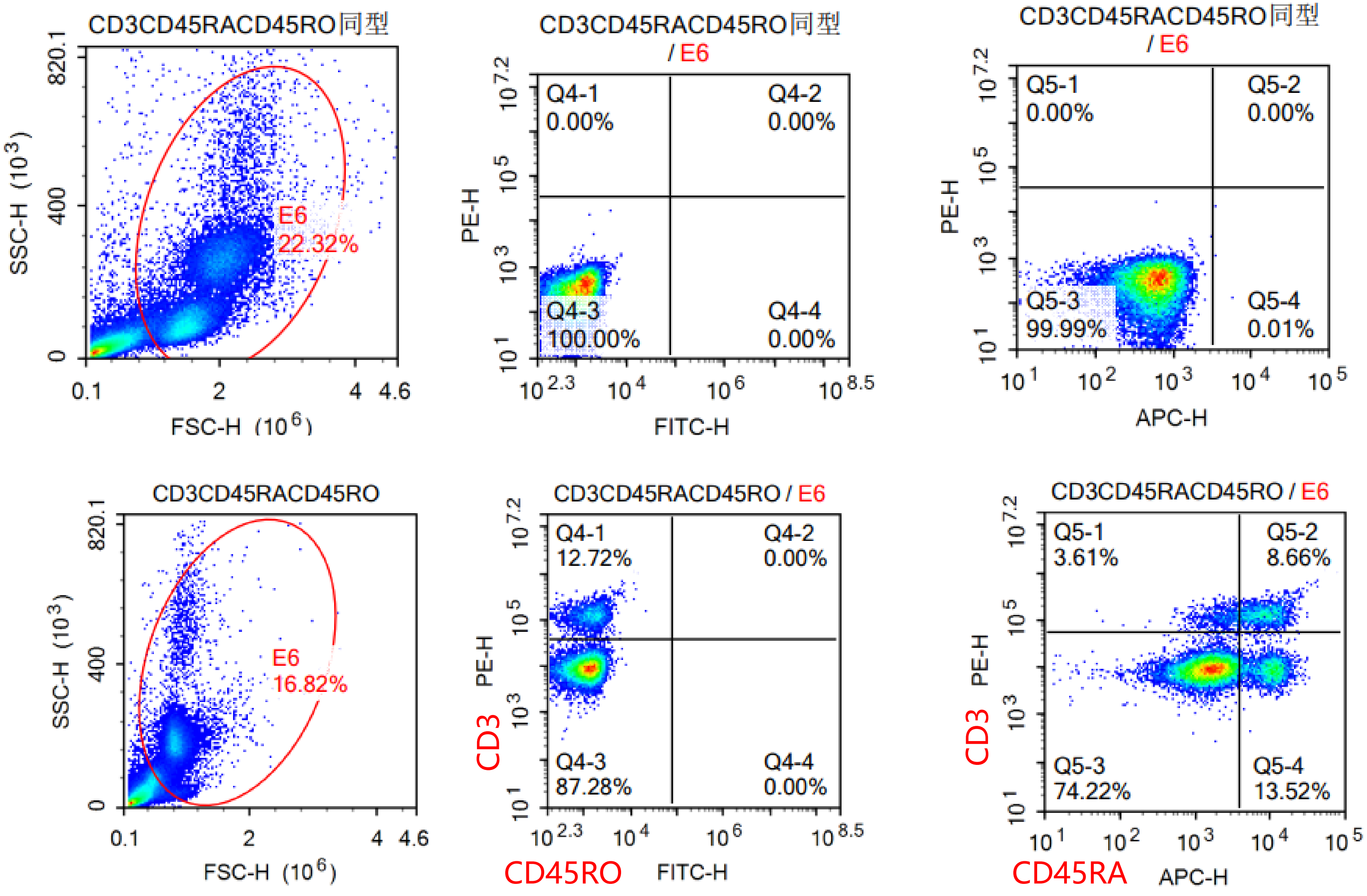

CD3+CD45RA+initial T cells account for 8.66%

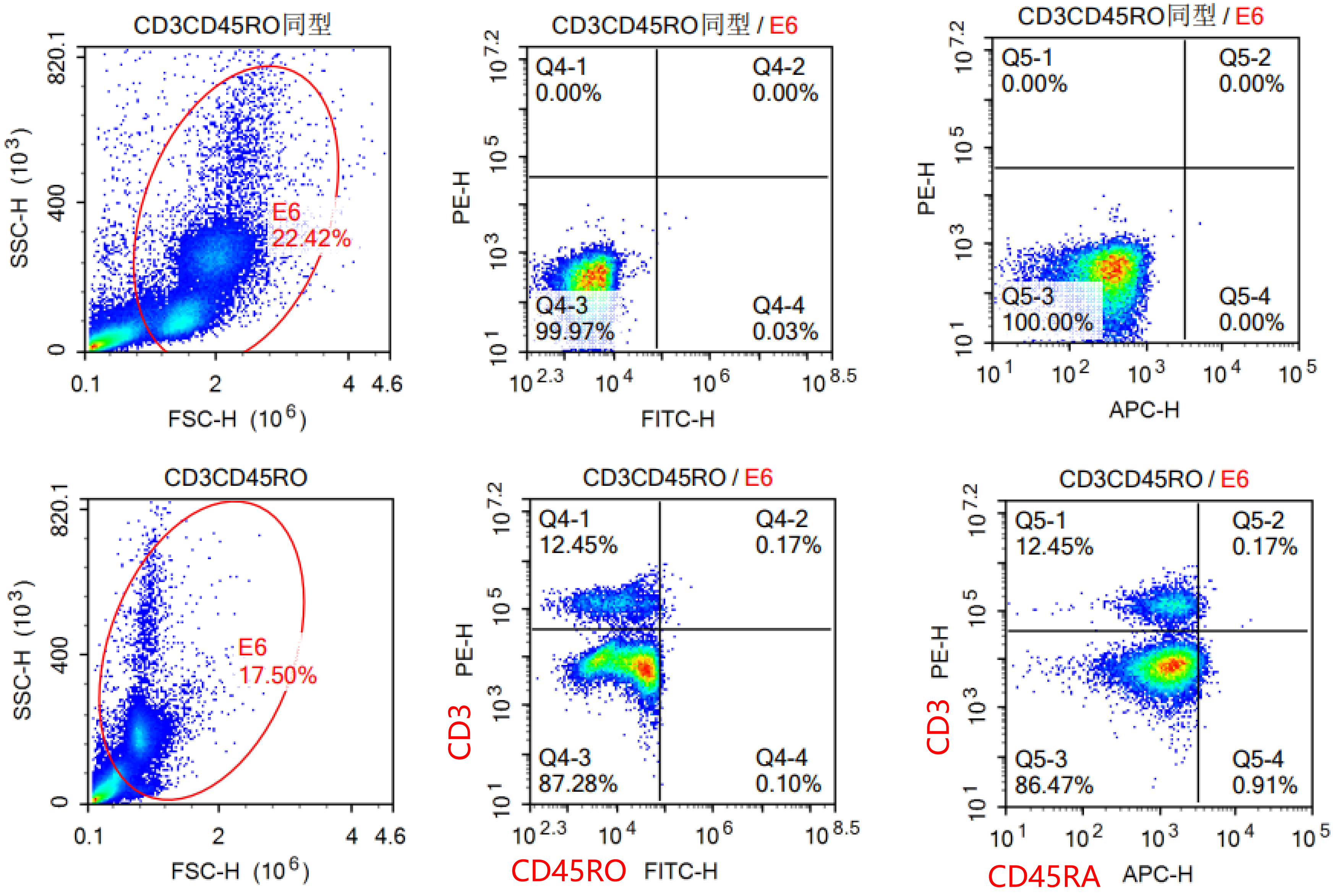

CD3+CD45RO+effector T/memory T cells account for 0.17%

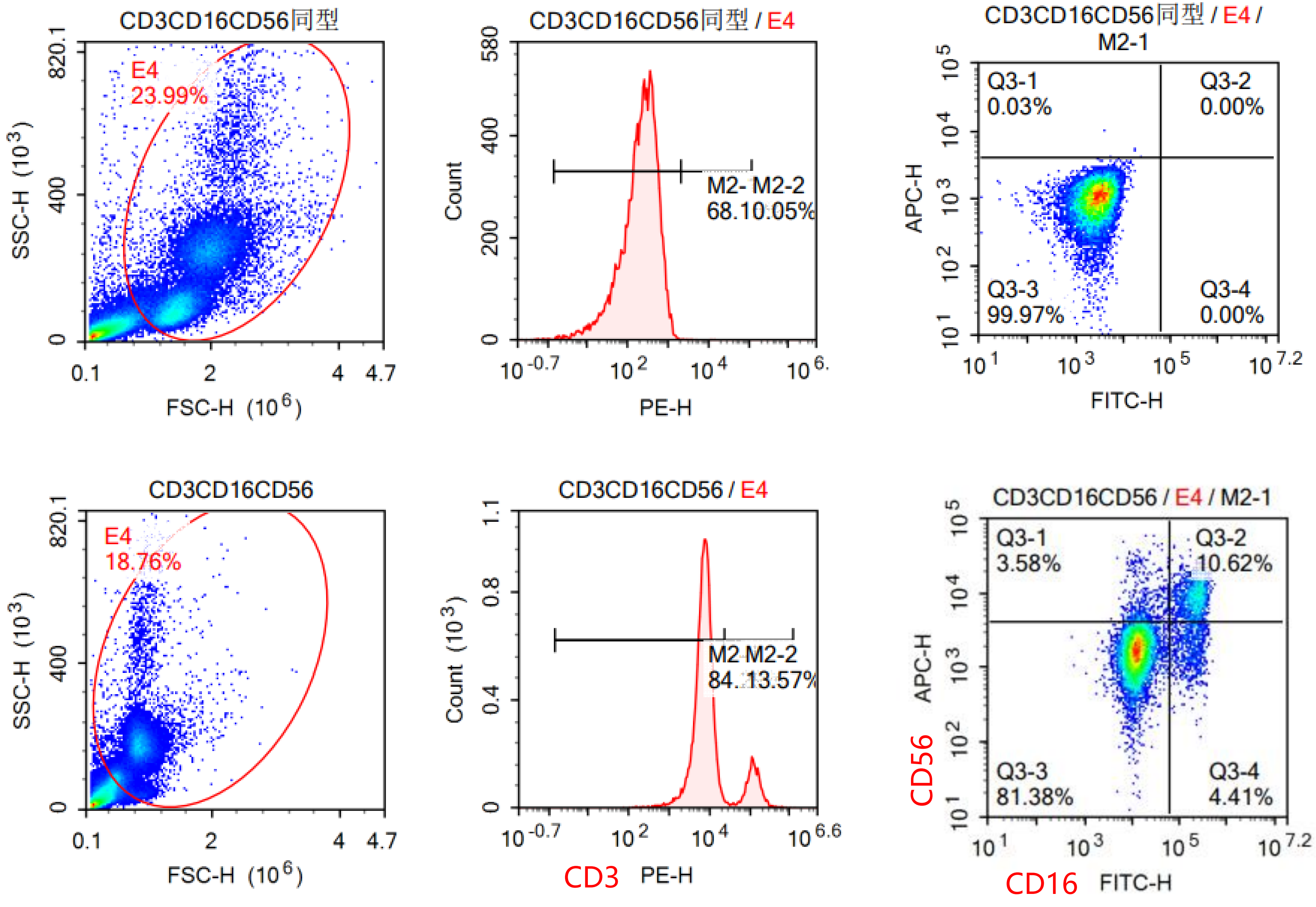

CD3-CD16+CD56+NK cells account for 8.92%
