# Supplementary material for: A Novel Cellular Therapy for Preterm Complications: Safety Profile of Allogeneic Cord Blood Mononuclear Cells in a Pilot Clinical Study
Source: Stem Cells Int. 2026 Aug 12;2026:9979914. doi: 10.1155/sci/9979914 (PMC13464116; doi:10.1155/sci/9979914)
Supplement: Supplementary file 2 [file SCI-2026-9979914-s004.pdf]

Molecular Genetic Disease Test Report
Institute of Clinical Genetics
Bayi Children's Hospital
Affiliated to General Hospital of Beijing Military Region, PLA

**Name**	JUHONG ZHIZI	**Gender**	Male	**Hospital No.**	859667	**Date Collected**	2016-01-11
**Sample No.**	1	**Age**		**Submitted by**		**Physician**	MEIYAPO

Analysis of Donor Cell Chimerism
After Allogeneic Cord Blood Mononuclear Cells Infusion

Test Conclusion

The proportion of donor-derived cells in the specimen is **0%**, indicating **complete recipient chimerism**.

Test Results

1. Donor/Recipient Allele Analysis

   Class I loci: D5S818, D21S11, D18S51, D6S1043, D16S539, D8S1179, D12S391, D2S1338, FGA

   Class II loci: D19S433, D3S1358, D13S317, D7S820, CSF1PO, Penta D, D2S441, vWA, Penta E, TH01

2. Donor Cell Chimerism Analysis

No donor-specific allele peaks were observed at all informative loci. No donor-derived cells were detected; the proportion of donor cells is **0%**.

Methods

1. Genomic DNA was extracted from the submitted specimen.
2. Multiplex amplification of genomic DNA was performed using an AB9700 PCR System and the Microreader™ 21Direct ID Kit.
3. Amplified products were analyzed by DNA sequencing on a 3130XL Genetic Analyzer with GeneMapper ID V3.2 software. Donor and recipient cells were distinguished based on genotype markers of **20 STR loci** (D19S433, D5S818, D21S11, D18S51, D6S1043, D3S1358, D13S317, D7S820, D16S539, CSF1PO, Penta D, D2S441, vWA, D8S1179, TPOX, Penta E, TH01, D12S391, D2S1338, FGA) and the sex-chromosome locus AMEL. The donor cell chimerism rate (ratio of donor to recipient cells post-transplantation) was calculated.
4. The detection sensitivity of this assay is **1%**.
5. Donor cell proportion ≥95%: **complete donor chimerism**
6. Donor cell proportion >5% and <95%: **mixed chimerism**
7. Donor cell proportion ≤5%: **complete recipient chimerism**

Note

This test result should be interpreted **in conjunction with other laboratory results and clinical manifestations**.

Reporter: Peng Wei

Reviewer: Wang Yan

Report Date: 2016-01-18

This result is solely valid for the submitted specimen only.

Molecular Genetic Disease Test Report
Institute of Clinical Genetics
Bayi Children's Hospital
Affiliated to General Hospital of Beijing Military Region, PLA

**Name**	XINCHONG XIAOZI	**Gender**	Male	**Hospital No.**	859150	**Date Collected**	2015-11-20
**Sample No.**	2	**Age**		**Submitted by**		**Physician**	MEIYAPO

Analysis of Donor Cell Chimerism
After Allogeneic Cord Blood Mononuclear Cells Infusion

Test Conclusion

The proportion of donor-derived cells in the specimen is **0%**, indicating **complete recipient chimerism**.

Test Results

1. Donor/Recipient Allele Analysis

Class I loci: D21S11、D18S51、D7S820、D16S539、D2S441、TPOX、2S1338、D8S1179

Class II loci:D19S433、D5S818、D6S1043、D3S1358、D13S317、PENTAD、vWA、PENTAE、TH01、D12S391、FGA

2. Donor Cell Chimerism Analysis

No donor-specific allele peaks were observed at all informative loci. No donor-derived cells were detected; the proportion of donor cells is **0%**.

Methods

1. Genomic DNA was extracted from the submitted specimen.
2. Multiplex amplification of genomic DNA was performed using an AB9700 PCR System and the Microreader™ 21Direct ID Kit.
3. Amplified products were analyzed by DNA sequencing on a 3130XL Genetic Analyzer with GeneMapper ID V3.2 software. Donor and recipient cells were distinguished based on genotype markers of **20 STR loci** (D19S433, D5S818, D21S11, D18S51, D6S1043, D3S1358, D13S317, D7S820, D16S539, CSF1PO, Penta D, D2S441, vWA, D8S1179, TPOX, Penta E, TH01, D12S391, D2S1338, FGA) and the sex-chromosome locus AMEL. The donor cell chimerism rate (ratio of donor to recipient cells post-transplantation) was calculated.
4. The detection sensitivity of this assay is **1%**.
5. Donor cell proportion ≥95%: **complete donor chimerism**
6. Donor cell proportion >5% and <95%: **mixed chimerism**
7. Donor cell proportion ≤5%: **complete recipient chimerism**

Note

This test result should be interpreted **in conjunction with other laboratory results and clinical manifestations**.

Reporter: Peng Wei

Reviewer: Wang Yan

Report Date: 2015-11-23

This result is solely valid for the submitted specimen only

.

Molecular Genetic Disease Test Report
Institute of Clinical Genetics
Bayi Children's Hospital
Affiliated to General Hospital of Beijing Military Region, PLA

**Name**	LIYULINGNV	**Gender**	Female	**Hospital No.**	860349	**Date Collected**	2015-12-11
**Sample No.**	3	**Age**		**Submitted by**		**Physician**	MEIYPO

Analysis of Donor Cell Chimerism
After Allogeneic Cord Blood Mononuclear Cells Infusion

Test Conclusion

The proportion of donor-derived cells in the specimen is **0%**, indicating **complete recipient chimerism**.

Test Results

1. Donor/Recipient Allele Analysis

Class I loci: D19S433、D5S818、D21S11、D18S51、D6S1043、D3S1358、D13S317、D7S820、D16S539、CSF1PO、Penta D、D2S441、vWA、D8S1179、Penta E、TH01、D12S391、D2S1338、FGA

Class II loci: TPOX

2. Donor Cell Chimerism Analysis

No donor-specific allele peaks were observed at all informative loci. No donor-derived cells were detected; the proportion of donor cells is **0%**.

Methods

1. Genomic DNA was extracted from the submitted specimen.
2. Multiplex amplification of genomic DNA was performed using an AB9700 PCR System and the Microreader™ 21Direct ID Kit.
3. Amplified products were analyzed by DNA sequencing on a 3130XL Genetic Analyzer with GeneMapper ID V3.2 software. Donor and recipient cells were distinguished based on genotype markers of **20 STR loci** (D19S433, D5S818, D21S11, D18S51, D6S1043, D3S1358, D13S317, D7S820, D16S539, CSF1PO, Penta D, D2S441, vWA, D8S1179, TPOX, Penta E, TH01, D12S391, D2S1338, FGA) and the sex-chromosome locus AMEL. The donor cell chimerism rate (ratio of donor to recipient cells post-transplantation) was calculated.
4. The detection sensitivity of this assay is **1%**.
5. Donor cell proportion ≥95%: **complete donor chimerism**
6. Donor cell proportion >5% and <95%: **mixed chimerism**
7. Donor cell proportion ≤5%: **complete recipient chimerism**

Note

This test result should be interpreted **in conjunction with other laboratory results and clinical manifestations**.

Reporter: Peng Wei

Reviewer: Wang Yan

This result is solely valid for the submitted specimen only.

Report Date: 2015-12-16

Molecular Genetic Disease Test Report
Institute of Clinical Genetics
Bayi Children's Hospital
Affiliated to General Hospital of Beijing Military Region, PLA

Name	KONG XINGCHENG	Gender	Male	Hospital No.	855194	Date Collected	2015-09-20
Sample No.	4	Age		Submitted by		Physician	MEIYAPO

Analysis of Donor Cell Chimerism
After Allogeneic Cord Blood Mononuclear Cells Infusion

Test Conclusion

The proportion of donor-derived cells in the specimen is **0%**, indicating **complete recipient chimerism**.

Test Results

1. Donor/Recipient Allele Analysis

   Class I loci: D18S51、D6S1043、D3S1358、PENTAD、vWA、D8S1179、PENTAE、FGA

   Class II loci: D19S433、D5S818、D21S11、D7S820、D16S539、TPOX、D12S391、2S1338

2. Donor Cell Chimerism Analysis

   No donor-specific allele peaks were observed at all informative loci. No donor-derived cells were detected; the proportion of donor cells is **0%**.

Methods

1. Genomic DNA was extracted from the submitted specimen.
2. Multiplex amplification of genomic DNA was performed using an AB9700 PCR System and the Microreader™ 21Direct ID Kit.
3. Amplified products were analyzed by DNA sequencing on a 3130XL Genetic Analyzer with GeneMapper ID V3.2 software. Donor and recipient cells were distinguished based on genotype markers of **20 STR loci** (D19S433, D5S818, D21S11, D18S51, D6S1043, D3S1358, D13S317, D7S820, D16S539, CSF1PO, Penta D, D2S441, vWA, D8S1179, TPOX, Penta E, TH01, D12S391, D2S1338, FGA) and the sex-chromosome locus AMEL. The donor cell chimerism rate (ratio of donor to recipient cells post-transplantation) was calculated.
4. The detection sensitivity of this assay is **1%**.
5. Donor cell proportion ≥95%: **complete donor chimerism**
6. Donor cell proportion >5% and <95%: **mixed chimerism**
7. Donor cell proportion ≤5%: **complete recipient chimerism**

Note

This test result should be interpreted **in conjunction with other laboratory results and clinical manifestations**.

Reporter: Peng Wei

Reviewer: Wang Yan

Report Date: 2015-09-23

This result is solely valid for the submitted specimen only.

Molecular Genetic Disease Test Report
Institute of Clinical Genetics
Bayi Children's Hospital
Affiliated to General Hospital of Beijing Military Region, PLA

**Name**	SUYANXIAOZI	**Gender**	Male	**Hospital No.**	860662	**Date Collected**	2016-01-27
**Sample No.**	5	**Age**		**Submitted by**		**Physician**	MEIYAPO

Analysis of Donor Cell Chimerism
After Allogeneic Cord Blood Mononuclear Cells Infusion

Test Conclusion

The proportion of donor-derived cells in the specimen is **0%**, indicating **complete recipient chimerism**.

Test Results

1. Donor/Recipient Allele Analysis

Class I loci: D19S433、D5S818、D21S11、D6S1043、D13S317、D16S539、CSF1PO、Penta D、D2S441、D8S1179、Penta E、D12S391、D2S1338、FGA

Class II loci: D3S1358、vWA、TH01

2. Donor Cell Chimerism Analysis

No donor-specific allele peaks were observed at all informative loci. No donor-derived cells were detected; the proportion of donor cells is **0%**.

Methods

1. Genomic DNA was extracted from the submitted specimen.
2. Multiplex amplification of genomic DNA was performed using an AB9700 PCR System and the Microreader™ 21Direct ID Kit.
3. Amplified products were analyzed by DNA sequencing on a 3130XL Genetic Analyzer with GeneMapper ID V3.2 software. Donor and recipient cells were distinguished based on genotype markers of **20 STR loci** (D19S433, D5S818, D21S11, D18S51, D6S1043, D3S1358, D13S317, D7S820, D16S539, CSF1PO, Penta D, D2S441, vWA, D8S1179, TPOX, Penta E, TH01, D12S391, D2S1338, FGA) and the sex-chromosome locus AMEL. The donor cell chimerism rate (ratio of donor to recipient cells post-transplantation) was calculated.
4. The detection sensitivity of this assay is **1%**.
5. Donor cell proportion ≥95%: **complete donor chimerism**
6. Donor cell proportion >5% and <95%: **mixed chimerism**
7. Donor cell proportion ≤5%: **complete recipient chimerism**

Note

This test result should be interpreted **in conjunction with other laboratory results and clinical manifestations**.

Reporter: Peng Wei

Reviewer: Wang Yan

Report Date: 2016-01-29

This result is solely valid for the submitted specimen only.

Molecular Genetic Disease Test Report
Institute of Clinical Genetics
Bayi Children's Hospital
Affiliated to General Hospital of Beijing Military Region, PLA

**Name**	SUYANDAZI	**Gender**	Male	**Hospital No.**	860661	**Date Collected**	2016-01-27
**Sample No.**	6	**Age**		**Submitted by**		**Physician**	MEIYAPO

Analysis of Donor Cell Chimerism
After Allogeneic Cord Blood Mononuclear Cells Infusion

Test Conclusion

The proportion of donor-derived cells in the specimen is **0%**, indicating **complete recipient chimerism**.

Test Results

1. Donor/Recipient Allele Analysis

Class I loci: D19S433、D5S818、D21S11、D6S1043、D3S1358、D13S317、D7S820、Penta D、D2S441、vWA、D8S1179、Penta E、D12S391、D2S1338、FGA

Class II loci: D18S51、D16S539、TPOX 、TH01

2. Donor Cell Chimerism Analysis

No donor-specific allele peaks were observed at all informative loci. No donor-derived cells were detected; the proportion of donor cells is **0%**.

Methods

1. Genomic DNA was extracted from the submitted specimen.
2. Multiplex amplification of genomic DNA was performed using an AB9700 PCR System and the Microreader™ 21Direct ID Kit.
3. Amplified products were analyzed by DNA sequencing on a 3130XL Genetic Analyzer with GeneMapper ID V3.2 software. Donor and recipient cells were distinguished based on genotype markers of **20 STR loci** (D19S433, D5S818, D21S11, D18S51, D6S1043, D3S1358, D13S317, D7S820, D16S539, CSF1PO, Penta D, D2S441, vWA, D8S1179, TPOX, Penta E, TH01, D12S391, D2S1338, FGA) and the sex-chromosome locus AMEL. The donor cell chimerism rate (ratio of donor to recipient cells post-transplantation) was calculated.
4. The detection sensitivity of this assay is **1%**.
5. Donor cell proportion ≥95%: **complete donor chimerism**
6. Donor cell proportion >5% and <95%: **mixed chimerism**
7. Donor cell proportion ≤5%: **complete recipient chimerism**

Note

This test result should be interpreted **in conjunction with other laboratory results and clinical manifestations**.

Reporter: Peng Wei

Reviewer: Wang Yan

Report Date: 2016-01-29

This result is solely valid for the submitted specimen only.

Molecular Genetic Disease Test Report
Institute of Clinical Genetics
Bayi Children's Hospital
Affiliated to General Hospital of Beijing Military Region, PLA

**Name**	WANGJINLINGZI	**Gender**	Male	**Hospital No.**	897132	**Date Collected**	2016-02-28
**Sample No.**	7	**Age**		**Submitted by**		**Physician**	MEIYAPO

Analysis of Donor Cell Chimerism
After Allogeneic Cord Blood Mononuclear Cells Infusion

Test Conclusion

The proportion of donor-derived cells in the specimen is **0%**, indicating **complete recipient chimerism**.

Test Results

1. Donor/Recipient Allele Analysis

   Class I loci: D21S11、D13S317、Penta D、vWA、Penta E、D12S391、D2S1338

   Class II loci: D19S433、D5S818、D6S1043、D3S1358、CSF1PO、D2S441、D8S1179、TH01、FGA

2. Donor Cell Chimerism Analysis

   No donor-specific allele peaks were observed at all informative loci. No donor-derived cells were detected; the proportion of donor cells is **0%**.

Methods

1. Genomic DNA was extracted from the submitted specimen.
2. Multiplex amplification of genomic DNA was performed using an AB9700 PCR System and the Microreader™ 21Direct ID Kit.
3. Amplified products were analyzed by DNA sequencing on a 3130XL Genetic Analyzer with GeneMapper ID V3.2 software. Donor and recipient cells were distinguished based on genotype markers of **20 STR loci** (D19S433, D5S818, D21S11, D18S51, D6S1043, D3S1358, D13S317, D7S820, D16S539, CSF1PO, Penta D, D2S441, vWA, D8S1179, TPOX, Penta E, TH01, D12S391, D2S1338, FGA) and the sex-chromosome locus AMEL. The donor cell chimerism rate (ratio of donor to recipient cells post-transplantation) was calculated.
4. The detection sensitivity of this assay is **1%**.
5. Donor cell proportion ≥95%: **complete donor chimerism**
6. Donor cell proportion >5% and <95%: **mixed chimerism**
7. Donor cell proportion ≤5%: **complete recipient chimerism**

Note

This test result should be interpreted **in conjunction with other laboratory results and clinical manifestations**.

Reporter: Peng Wei

Reviewer: Wang Yan

Report Date: 2016-03-02

This result is solely valid for the submitted specimen only.

Molecular Genetic Disease Test Report
Institute of Clinical Genetics
Bayi Children's Hospital
Affiliated to General Hospital of Beijing Military Region, PLA

**Name**	YUANCHUNHUA XIAO	**Gender**	Male	**Hospital No.**	859047	**Date Collected**	2015-09-30
**Sample No.**	8	**Age**		**Submitted by**		**Physician**	MEIYAPO

Analysis of Donor Cell Chimerism
After Allogeneic Cord Blood Mononuclear Cells Infusion

Test Conclusion

The proportion of donor-derived cells in the specimen is **0%**, indicating **complete recipient chimerism**.

Test Results

1. Donor/Recipient Allele Analysis

   Class I loci: D19S433、D5S818、D6S1043、D16S539、PENTAD、D12S391、2S1338、FGA

   Class II loci: D18S51、D3S1358、D13S317、D7S820、CSF1PO、D2S441、vWA、D8S1179、PENTAE

2. Donor Cell Chimerism Analysis

   No donor-specific allele peaks were observed at all informative loci. No donor-derived cells were detected; the proportion of donor cells is **0%**.

Methods

1. Genomic DNA was extracted from the submitted specimen.
2. Multiplex amplification of genomic DNA was performed using an AB9700 PCR System and the Microreader™ 21Direct ID Kit.
3. Amplified products were analyzed by DNA sequencing on a 3130XL Genetic Analyzer with GeneMapper ID V3.2 software. Donor and recipient cells were distinguished based on genotype markers of **20 STR loci** (D19S433, D5S818, D21S11, D18S51, D6S1043, D3S1358, D13S317, D7S820, D16S539, CSF1PO, Penta D, D2S441, vWA, D8S1179, TPOX, Penta E, TH01, D12S391, D2S1338, FGA) and the sex-chromosome locus AMEL. The donor cell chimerism rate (ratio of donor to recipient cells post-transplantation) was calculated.
4. The detection sensitivity of this assay is **1%**.
5. Donor cell proportion ≥95%: **complete donor chimerism**
6. Donor cell proportion >5% and <95%: **mixed chimerism**
7. Donor cell proportion ≤5%: **complete recipient chimerism**

Note

This test result should be interpreted **in conjunction with other laboratory results and clinical manifestations**.

Reporter: Peng Wei

Reviewer: Wang Yan

Report Date: 2015-10-08

This result is solely valid for the submitted specimen only.

Molecular Genetic Disease Test Report
Institute of Clinical Genetics
Bayi Children's Hospital
Affiliated to General Hospital of Beijing Military Region, PLA

**Name**	YUANCHUNHUADA	**Gender**	Male	**Hospital No.**	859048	**Date Collected**	2015-10-13
**Sample No.**	9	**Age**		**Submitted by**		**Physician**	MEIYAPO

Analysis of Donor Cell Chimerism
After Allogeneic Cord Blood Mononuclear Cells Infusion

Test Conclusion

The proportion of donor-derived cells in the specimen is **0%**, indicating **complete recipient chimerism**.

Test Results

1. Donor/Recipient Allele Analysis

Class I loci: D13S317、D7S820、PENTAD、PENTAE、2S1338

Class II loci: D21S11、D18S51、D6S1043、D3S1358、CSF1PO、D2S441、vWA、D8S1179、TPOX、TH01、FGA

2. Donor Cell Chimerism Analysis

No donor-specific allele peaks were observed at all informative loci. No donor-derived cells were detected; the proportion of donor cells is **0%**.

Methods

1. Genomic DNA was extracted from the submitted specimen.
2. Multiplex amplification of genomic DNA was performed using an AB9700 PCR System and the Microreader™ 21Direct ID Kit.
3. Amplified products were analyzed by DNA sequencing on a 3130XL Genetic Analyzer with GeneMapper ID V3.2 software. Donor and recipient cells were distinguished based on genotype markers of **20 STR loci** (D19S433, D5S818, D21S11, D18S51, D6S1043, D3S1358, D13S317, D7S820, D16S539, CSF1PO, Penta D, D2S441, vWA, D8S1179, TPOX, Penta E, TH01, D12S391, D2S1338, FGA) and the sex-chromosome locus AMEL. The donor cell chimerism rate (ratio of donor to recipient cells post-transplantation) was calculated.
4. The detection sensitivity of this assay is **1%**.
5. Donor cell proportion ≥95%: **complete donor chimerism**
6. Donor cell proportion >5% and <95%: **mixed chimerism**
7. Donor cell proportion ≤5%: **complete recipient chimerism**

Note

This test result should be interpreted **in conjunction with other laboratory results and clinical manifestations**.

Reporter: Peng Wei

Reviewer: Wang Yan

Report Date: 2015-10-13

This result is solely valid for the submitted specimen only.

Molecular Genetic Disease Test Report
Institute of Clinical Genetics
Bayi Children's Hospital
Affiliated to General Hospital of Beijing Military Region, PLA

**Name**	LINANZHIZI	**Gender**	Male	**Hospital No.**	855094	**Date Collected**	2015-09-07
**Sample No.**	10	**Age**		**Submitted by**		**Physician**	MEIYAPO

Analysis of Donor Cell Chimerism
After Allogeneic Cord Blood Mononuclear Cells Infusion

Test Conclusion

The proportion of donor-derived cells in the specimen is **0%**, indicating **complete recipient chimerism**.

Test Results

1. Donor/Recipient Allele Analysis

Class I loci: D19S433、D21S11、D18S51、D6S1043、D3S1358、D16S539、CSF1PO、Penta D、D2S441、vWA、D8S1179、Penta E、TH01、D12S391、D2S1338、FGA

Class II loci: D13S317、D7S820、TPOX

2. Donor Cell Chimerism Analysis

No donor-specific allele peaks were observed at all informative loci. No donor-derived cells were detected; the proportion of donor cells is **0%**.

Methods

1. Genomic DNA was extracted from the submitted specimen.
2. Multiplex amplification of genomic DNA was performed using an AB9700 PCR System and the Microreader™ 21Direct ID Kit.
3. Amplified products were analyzed by DNA sequencing on a 3130XL Genetic Analyzer with GeneMapper ID V3.2 software. Donor and recipient cells were distinguished based on genotype markers of **20 STR loci** (D19S433, D5S818, D21S11, D18S51, D6S1043, D3S1358, D13S317, D7S820, D16S539, CSF1PO, Penta D, D2S441, vWA, D8S1179, TPOX, Penta E, TH01, D12S391, D2S1338, FGA) and the sex-chromosome locus AMEL. The donor cell chimerism rate (ratio of donor to recipient cells post-transplantation) was calculated.
4. The detection sensitivity of this assay is **1%**.
5. Donor cell proportion ≥95%: **complete donor chimerism**
6. Donor cell proportion >5% and <95%: **mixed chimerism**
7. Donor cell proportion ≤5%: **complete recipient chimerism**

Note

This test result should be interpreted **in conjunction with other laboratory results and clinical manifestations**.

Reporter: Peng Wei

Reviewer: Wang Yan

Report Date: 2015-09-09

This result is solely valid for the submitted specimen only.
