# Supplementary material for: A Novel Cellular Therapy for Preterm Complications: Safety Profile of Allogeneic Cord Blood Mononuclear Cells in a Pilot Clinical Study
Source: Stem Cells Int. 2026 Aug 12;2026:9979914. doi: 10.1155/sci/9979914 (PMC13464116; doi:10.1155/sci/9979914)
Supplement: Supplementary file 3 [file SCI-2026-9979914-s001.docx]

Supplemental Table 2. Characteristics of human umbilical cord blood-derived mononuclear cells (hUCB-MNCs)

Panel A: Cord blood characterization and microbiological safety testing

| **Patient ID** | **Cord blood ID** | **TNC(×10⁸)** | **CD34⁺(%)** | **CD34⁺(×10⁶)** | **CFU-GM(×10⁶)** | **Bacterialculture** | **HBsAg** | **Anti-HCV** | **Anti-HIV(I/II)** | **Syphilis** |
| --- | --- | --- | --- | --- | --- | --- | --- | --- | --- | --- |
| **Patient 1** | JX20140820011 | 8.94 | 0.56 | 5.00 | 1.340 | Negative | Negative | Negative | Negative | Negative |
| **Patient 2** | JX20140620025 | 9.81 | 0.42 | 4.12 | 0.961 | Negative | Negative | Negative | Negative | Negative |
| **Patient 3** | JX20140514016 | 15.21 | 0.24 | 3.65 | 1.422 | Negative | Negative | Negative | Negative | Negative |
| **Patient 4** | 2005100501 | 8.18 | 0.84 | 6.87 | 2.764 | Negative | Negative | Negative | Negative | Negative |
| **Patient 5** | 2006121502 | 11.54 | 0.30 | 3.46 | 0.831 | Negative | Negative | Negative | Negative | Negative |
| **Patient 6** | JX20140302006 | 9.88 | 0.44 | 4.35 | 0.699 | Negative | Negative | Negative | Negative | Negative |
| **Patient 7** | 2012022104 | 9.75 | 0.43 | 4.19 | 1.969 | Negative | Negative | Negative | Negative | Negative |
| **Patient 8** | 20121120006 | 8.54 | 0.65 | 5.55 | 1.827 | Negative | Negative | Negative | Negative | Negative |
| **Patient 9** | 2005041401 | 9.22 | 0.37 | 3.41 | 1.715 | Negative | Negative | Negative | Negative | Negative |
| **Patient 10** | JX20141021008 | 24.35 | 0.22 | 5.35 | 2.191 | Negative | Negative | Negative | Negative | Negative |

Panel B: Infused cell product parameters

| Patient ID | **MNC concentration**  **(×10^8^/L)** | **Infused volume(mL)** | **Total infused MNC (×10^6^)** | **CD34⁺(×10⁶)** | **CD34⁺ recovery(%)** | **Total infused MNC (×10^6^/kg)** | **Body weight at infusion (kg)** |
| --- | --- | --- | --- | --- | --- | --- | --- |
| Patient 1 | 4.76 | 10.5 | 5 | 2.30 | 46.0 | 3.35 | 1.49 |
| Patient 2 | 4.14 | 10.0 | 4.14 | 0.77 | 18.6 | 2.76 | 1.5 |
| Patient 3 | 3.04 | 12.0 | 3.65 | 1.15 | 31.5 | 2.35 | 1.55 |
| Patient 4 | 5.28 | 13.0 | 6.87 | 3.71 | 54.0 | 3.44 | 2.0 |
| Patient 5 | 2.46 | 14.0 | 3.45 | 1.20 | 34.7 | 3.22 | 1.07 |
| Patient 6 | 3.31 | 13.0 | 4.30 | 2.00 | 46.0 | 4.05 | 1.06 |
| Patient 7 | 4.18 | 10.0 | 4.18 | 2.93 | 70.0 | 4.18 | 1.0 |
| Patient 8 | 5.50 | 10.0 | 5.55 | 3.07 | 55.3 | 5.55 | 1.0 |
| Patient 9 | 3.09 | 11 | 3.4 | 3.28 | 96.1 | 2.62 | 1.3 |
| Patient 10 | 4.12 | 13.0 | 5.35 | 3.02 | 56.4 | 3.57 | 1.5 |

Abbreviations: TNC, total nucleated cells; CFU-GM, colony-forming unit granulocyte-macrophage; HBsAg, hepatitis B surface antigen; Anti-HCV, antibody to hepatitis C virus;

Anti-HIV, antibody to human immunodeficiency virus; MNC, mononuclear cell.
