# Supplementary material for: A Novel Cellular Therapy for Preterm Complications: Safety Profile of Allogeneic Cord Blood Mononuclear Cells in a Pilot Clinical Study
Source: Stem Cells Int. 2026 Aug 12;2026:9979914. doi: 10.1155/sci/9979914 (PMC13464116; doi:10.1155/sci/9979914)
Supplement: Supplementary file 4 [file SCI-2026-9979914-s003.docx]

**ELISA kit test report**

# 1.Experimental Materials

### 1.1.Experimental instruments

**Table1 The main equipment involved in the experiment**

| **Name** | **Model** | **Manufacturer** |
| --- | --- | --- |
| **微型高速离心机** | C2500-R-230V | USA Labnet |
| 电热恒温培养箱 | ICV-450 | Japan ASONE |
| 全自动酶标仪 | Multiskan MK3 | Thermo scientific |

### 1.2.Main experimental reagents

**Table 2-1 The main reagents involved in the experiment**

| **Reagents Name** | **Item number** | **Manufacturer** |
| --- | --- | --- |
| Human tumor necrosis factor-α(TNF-α)  Enzyme linked immunosorbent assay kit | E-EL-H0109c | Eli Lilly Biotechnology Co., Ltd |
| Human hepatocyte growth factor(HGF)  Enzyme linked immunosorbent assay kit | E-EL-H0084c | Eli Lilly Biotechnology Co., Ltd |
| Human matrix metalloproteinase-9(MMP-9)  Enzyme linked immunosorbent assay kit | E-EL-H1451c | Eli Lilly Biotechnology Co., Ltd |
| human vascular endothelial growth factor  (VEGF)  Enzyme linked immunosorbent assay kit | E-EL-H0111c | Eli Lilly Biotechnology Co., Ltd |
| Human transforming growth factor-β1  (TGF-β1)  Enzyme linked immunosorbent assay kit | E-EL-H0110c | Eli Lilly Biotechnology Co., Ltd |
| Human interleukin-1(IL-1α)  Enzyme linked immunosorbent assay kit | E-EL-H0088c | Eli Lilly Biotechnology Co., Ltd |
| Human interleukin-6(IL-6)  Enzyme linked immunosorbent assay kit | E-EL-H0102c | Eli Lilly Biotechnology Co., Ltd |
| Human interleukin-8(IL-8)  Enzyme linked immunosorbent assay kit | E-EL-H0048c | Eli Lilly Biotechnology Co., Ltd |
| Human interleukin-10(IL-10)  Enzyme linked immunosorbent assay kit | E-EL-H0103c | Eli Lilly Biotechnology Co., Ltd |

# 2. Experimental procedure

### 2.1. Sample processing

Serum sample (TNF-α, HGF, VEGF, MMP9, IL-1, IL-6, IL-8, and IL-10).

Heating activation(TGF-β1): Dilute the sample 20 times with standard and sample diluent, place it in an 80 ℃ water bath for 8 minutes, remove and cool for 5 minutes, and test within 2 hours.

### 2.2. Experimental procedure

Before the experiment, all reagents should be equilibrated to room temperature; When preparing reagents or samples, they should be fully mixed and bubbling should be avoided as much as possible.

2.2.1. Sample addition: Set up blank wells, standard wells, and sample wells for testing. Add 100μL of standard and sample diluent to the blank well, and add 100μL of standard or sample to the remaining wells. Take care not to introduce air bubbles. When adding the sample, place it at the bottom of the microplate, avoiding contact with the well walls. Gently shake to mix. Cover the microplate with a film and incubate at 37℃ for 90 minutes. To ensure the validity of the experimental results, please use a new standard solution for each experiment.

2.2.2. Discard the liquid, spin dry, and do not wash. Add 100 μL of biotinylated antibody working solution to each well (prepared within 15 minutes before use), cover the microplate with a cover slip, and incubate at 37°C for 1 hour.

2.2.3. Discard the liquid in the well, shake the plate dry, wash it 3 times, soaking for 1-2 minutes each time, with approximately 350 μL/well. Then, shake the plate dry and gently tap it on absorbent paper to dry the liquid in the well.

2.2.4. Add 100 μ L of enzyme conjugate working solution (prepared within 15 minutes before use) to each well, cover with film, and incubate at 37 ℃ for 30 minutes.

2.2.5. Discard the liquid in the hole, shake dry, and wash the plate 5 times using the same method as step 3.

2.2.6. Add 90 μ L of substrate solution (TMB) to each well, and incubate the enzyme-linked immunosorbent assay (ELISA) plate with a film at 37 ° C in the dark for about 15 minutes (shorten or prolong according to the actual color development situation, but not exceed 30 minutes). When a significant gradient appears in the standard well, it can be terminated.

2.2.7. Add 50 μ L of termination solution to each well to terminate the reaction, at which point the blue color will turn yellow. The order of adding termination solution should be as consistent as possible with the order of adding substrate solution.

2.2.8. Immediately measure the optical density (OD value) of each well using an enzyme-linked immunosorbent assay (ELISA) reader at a wavelength of 450nm. The power of the enzyme-linked immunosorbent assay (ELISA) reader should be turned on in advance, the instrument should be preheated, and the detection program should be set up.

# 3. Experimental results
