# Supplementary material for: A Novel Cellular Therapy for Preterm Complications: Safety Profile of Allogeneic Cord Blood Mononuclear Cells in a Pilot Clinical Study
Source: Stem Cells Int. 2026 Aug 12;2026:9979914. doi: 10.1155/sci/9979914 (PMC13464116; doi:10.1155/sci/9979914)
Supplement: Supplementary file 5 [file SCI-2026-9979914-s005.docx]

Supplemental table1 Adverse Events

| **Patient lD** | **AE Type** | **Possibly drug**  **related adverse**  **events** | **CTCAE Grade** | **Age at Onset (Post-infusion)** | **Onset Time** | **Duration** | **Management Measures** | **Outcome** |
| --- | --- | --- | --- | --- | --- | --- | --- | --- |
| **Patient 1** | No adverse event | N/A | N/A | N/A | N/A | N/A | N/A | N/A |
| **Patient 2** | Community-acquired pneumonia | unrelated | 2 | 6-month | 6-month follow-up | 7 days | Standard empirical antibiotic therapy; nasal cannula oxygen support (FiO2 30%); symptomatic care (antipyretic, cough relieving) | Complete recovery after 7 days of treatment; no residual respiratory symptoms |
| **Patient 3** | Transient  hypertension | Possibly related to cell suspension volume load; unrelated to hUCB-MNCs immunogenicity | 1 | 1h | Day 0  (infusion day) | 1h | Close vital sign monitoring;  no pharmacological intervention | Complete resolution within 1 h; no recurrence |
| **Patient 4** | Transient  hypertension | Possibly related to cell suspension volume load; unrelated to hUCB-MNCs immunogenicity | 1 | 2h | Day 0  (infusion day) | 45min | Close vital sign monitoring;  no pharmacological intervention | Complete resolution within 1 h; no recurrence |
| **Patient 5** | No adverse event | N/A | N/A | N/A | N/A | N/A | N/A | No AE occurrence during the entire follow-up period |
| **Patient 6** | Cerebral palsy | unrelated | 3 | 1st follow-up visit | 12-month follow-up | Persistent | Standard rehabilitation therapy (gross motor/fine motor training, language intervention, occupational therapy); regular neurological follow-up | Stable condition, no progression of neurological deficit; partial motor function improvement after rehabilitation |
| **Patient 7** | No adverse event | N/A | N/A | N/A | N/A | N/A | N/A | No AE  Occurrence in hospital |
| **Patient 8** | No adverse event | N/A | N/A | N/A | N/A | N/A | N/A | No AE  Occurrence in hospital |
| **Patient 9** | No adverse event | N/A | N/A | N/A | N/A | N/A | N/A | No AE  Occurrence in hospital |
| **Patient 10** | No adverse event | N/A | N/A | N/A | N/A | N/A | N/A | No AE  Occurrence in hospital |

AEs were adjudicated by the study team and classified based on Common Terminology Criteria for AEs version 5.0
