# Supplementary material for: A Novel Cellular Therapy for Preterm Complications: Safety Profile of Allogeneic Cord Blood Mononuclear Cells in a Pilot Clinical Study
Source: Stem Cells Int. 2026 Aug 12;2026:9979914. doi: 10.1155/sci/9979914 (PMC13464116; doi:10.1155/sci/9979914)
Supplement: Supplementary file 6 [file SCI-2026-9979914-s006.docx]

**Supplemental Table 3 Expression profiles of inflammatory factors and growth factors in peripheral blood at baseline and after hUCB-MNCs infusion**

| **Index** | **Mean difference** | **Median difference** | **SD** | **SE mean** | **95% CI of difference** | **t/z** | **df** | **P value** |
| --- | --- | --- | --- | --- | --- | --- | --- | --- |
| IL‑1α | 90.026 | - | 49.036 | 15.506 | 54.948, 125.104 | 5.806 | 9 | <0.001 |
| IL‑6 | 35.053 | - | 14.050 | 4.443 | 25.002, 45.104 | 7.889 | 9 | <0.001 |
| IL‑8 | 117.594 | - | 19.004 | 6.009 | 104.000, 131.188 | 19.568 | 9 | <0.001 |
| IL‑10 | 67.146 | - | 44.972 | 14.221 | 34.975, 99.317 | 4.721 | 9 | 0.001 |
| VEGF | 145.707 | - | 101.122 | 31.977 | 73.369, 218.045 | 4.557 | 9 | 0.001 |
| HGF | −193.705 | - | 56.652 | 17.915 | −234.231, −153.179 | −10.812 | 9 | <0.001 |
| MMP9 | 316.277 | - | 157.430 | 49.784 | 203.658, 428.896 | 6.353 | 9 | <0.001 |
| IFN‑α | — | −41.930 | - | - | −52.790, −14.600 | −2.475 | - | 0.013 |
| TGF‑β1 | — | −7718.130 | - | - | −18194.900, −1389.025 | −2.701 | - | 0.007 |
