# Supplementary material for: A Novel Cellular Therapy for Preterm Complications: Safety Profile of Allogeneic Cord Blood Mononuclear Cells in a Pilot Clinical Study
Source: Stem Cells Int. 2026 Aug 12;2026:9979914. doi: 10.1155/sci/9979914 (PMC13464116; doi:10.1155/sci/9979914)
Supplement: Supplementary file 7 [file SCI-2026-9979914-s007.docx]

**Supplemental materials 2**

**Assessment of follow-up**

This follow-up study protocol was approved by the ethics committee of the Seventh Medical Center of PLA General Hospital, Beijing, China (No.2020-037) on May 18, and the study was registered on Chinese Clinical Trials.gov (NO. ChiCTR – OPN - 15006932). Informed consent for the study was obtained from the parents of each child after review with the principal investigator or study staff.

**Participants and methods**

Six infants discharged alive were prospectively followed up until 24 months of corrected age (CA). The primary goal was to determine the long-term safety of hUCB-MNCs transplantation in the enrolled infants up to 24 months of CA. Safety was defined as the absence of treatment-related serious adverse events, which defined as an untoward medical occurrence that resulted in death, or life-threatening, required inpatient hospitalization or prolongation of existing hospitalization, or resulted in persistent or significant disability. The secondary goal was to evaluate the potential long-term effects of hUCB-MNCs infusion on growth, respiratory outcomes, and neurodevelopmental outcomes up to 24 months of CA. Infants were evaluated at 12months and 24 months of CA in the Neonatal Follow-up Clinic of Health. At each visit, infants were examined with standard outcome assessments, including ascertainment of growth, respiratory health status, and neurodevelopmental outcomes by using a standardized follow-up interview, growth measurements, and thorough physical and neurologic examinations. In the standardized interview with parents, all medication use (including bronchodilators, inhaled/systemic steroid and home oxygen therapy), the occurrence of wheezing or whistling sounds in the chest, and rehospitalization since the last visit were documented. The infants’ height, body weight, and head circumference were measured for growth assessment, and these values were according to the database for 2009 Chinese National Growth Charts [14]. Catch-up growth was defined as >10th percentile weight, height, or head circumference according to the 2009 Chinese National Growth Charts. At 12~24 months of CA, the neurodevelopmental outcome was evaluated with the Neuropsychological Development Sale (NDS) for Children, which made by Children’s Hospital Capital Institute of Pediatrics of China. Neuropsydevelopmental delay was defined as NDS score <70. Blindness was defined as no useful vision in either eye. Deafness was defined as requiring hearing amplification aids in both ears. Cerebaral palsy was defined as a Palisano gross motor function score[15]≥2. The score on the NDS was based on the parent’s report of language skill attainment and the child’s performance with the administered items. The scores from the NDS were detailed in the supplemental materials.

H. Li, X.-N. Ji Cy Fau - Zong, Y.-Q. Zong Xn Fau - Zhang, Y.Q. Zhang, [Height and weight standardized growth charts for Chinese children and adolescents aged 0 to 18 years].

R.A.-O. Palisano, L. Avery, J.A.-O. Gorter, B. Galuppi, S.W. McCoy, Stability of the Gross Motor Function Classification System, Manual Ability Classification System, and Communication Function Classification System.

eFigure 3
